# Preoperative lipid concentrations and 28-day mortality after cardiac surgery with cardiopulmonary bypass: a retrospective analysis of a prospectively maintained registry

**DOI:** 10.1016/j.clinsp.2026.101224

**Published:** 2026-11-03

**Authors:** Christian de Tymowski, Cécile Echard, Anne Boutten, Patrick Nataf, Marylou Para, Bernard Iung, Olivier Meilhac, Stefan Andrei, Dan Longrois, Philippe Montravers, Sophie Provenchère, Sébastien Tanaka

**Affiliations:** a Department of Anesthesiology, Hôpital Louis Mourier, DMU PARABOLAssistance Publique-Hôpitaux de Paris (AP-HP), Colombes, France; b Université Paris Cité, Paris, France; c Department of Anesthesiology and Critical Care Medicine, Assistance Publique-Hôpitaux de Paris (AP-HP), Bichat-Claude Bernard Hospital, Paris, France; d French Institute of Health and Medical Research (INSERM) U1149, Center for Research on Inflammation, Paris, France; e Biochemistry Department, Assistance Publique-Hôpitaux de Paris (AP-HP), Bichat-Claude Bernard Hospital, Paris, France; f Cardiac Surgery Department, Assistance Publique-Hôpitaux de Paris (AP-HP), Bichat-Claude Bernard Hospital, Paris, France; g Laboratory for Vascular Translational Science, French Institute of Health and Medical Research (INSERM) U1148, Paris, France; h Cardiology Department, Assistance Publique-Hôpitaux de Paris (AP-HP), Bichat-Claude Bernard Hospital, Paris, France; i French Institute of Health and Medical Research (INSERM), U1188 Diabetes Atherothrombosis Réunion Indian Ocean (DéTROI), Réunion Island University, Saint Pierre de La Réunion, France; j Réunion Island University, Saint Pierre de La Réunion, France

**Keywords:** Lipid profile, LDL, HDL, Mortality, Cardiac surgery

## Abstract

**Background:**

Low cholesterol is associated with poor outcomes in acute illness, although low LDL-Cholesterol (LDL-C) is a therapeutic target in cardiovascular disease. We assessed whether preoperative lipid concentrations were associated with 28-day mortality after cardiac surgery with Cardiopulmonary Bypass (CPB).

**Methods:**

We retrospectively analysed a prospectively maintained single-centre registry of cardiac surgery with CPB (April 2013–December 2023), linked to preoperative lipid measurements and national death records. LDL-C was the primary exposure. Models were adjusted for EuroSCORE 2, statin use, infective endocarditis, urgent surgery, and critical preoperative state. Secondary analyses were corrected for multiplicity (Benjamini-Hochberg).

**Results:**

Of 10,088 patients, 71 were excluded (reoperation within one year or duplicate record) and 1,986 of the remaining 10,017 had a preoperative lipid panel; 64 (3.2%) died within 28-days. In unadjusted analyses, lower Total Cholesterol (TC), HDL-C and LDL-C were associated with mortality. After adjustment, LDL-C was no longer associated with mortality (adjusted odds ratio = 0.803, 95% CI 0.595–1.084; p = 0.152). TC retained a borderline association (0.777, 0.607–0.994; p = 0.045), not surviving correction (*q* = 0.173). Lipid parameters discriminated poorly (areas under the curve 0.57–0.62) and added nothing to EuroSCORE 2.

**Conclusions:**

After adjustment for illness severity, preoperative LDL-C was not associated with 28-day mortality, and lipids provided no prognostic information beyond EuroSCORE 2. Very low TC may primarily reflect illness severity rather than an independent causal risk factor, and these data provide no basis for modifying perioperative lipid-lowering therapy.

## Highlights

• Low preoperative cholesterol was linked to 28-day mortality only before adjustment.
• After adjustment for illness severity, LDL-C was not associated with 28-day mortality.
• Total cholesterol showed only a borderline association with 28-day mortality.
• Lipid concentrations added no prognostic information beyond EuroSCORE 2.
• Low total cholesterol is best read as a marker of preoperative illness severity.

## Introduction

Cholesterol is one of the most extensively studied prognostic markers in cardiovascular medicine. Since the Framingham cohort, the concentrations of Low-Density Lipoprotein Cholesterol (LDL-C) and High-Density Lipoprotein Cholesterol (HDL-C) have been associated with the incidence of cardiovascular events,^1^^,^^2^ and LDL-C is now regarded as a causal factor of atherosclerotic cardiovascular disease.^3^ Consequently, international guidelines recommend lowering LDL-C in patients at high or very high cardiovascular risk, with a target below 1.4 mmoL/L in the latter group, mainly through statin therapy.^4^ The benefit of this strategy is, however, conditional on the starting concentration: in a meta-analysis of 34 randomized trials, LDL-C lowering reduced all-cause mortality only when baseline LDL-C was 2.6 mmoL/L (100 mg/dL) or higher, with a benefit increasing with the baseline value and no demonstrable reduction below this level.^5^ Low LDL-C is therefore, in chronic conditions, a therapeutic objective rather than a warning sign, but lowering an already low LDL-C has not been shown to improve survival.

In the setting of an acute insult, however, low cholesterol concentrations have repeatedly been associated with a poor prognosis. Total Cholesterol (TC), HDL-C and LDL-C fall as part of the acute-phase response to inflammation, sepsis, hepatic dysfunction and malnutrition.^6^ In sepsis, low admission concentrations of TC, HDL-C and LDL-C, but not of Triglycerides (TG), are associated with mortality, and a recent meta-analysis framed hypocholesterolaemia primarily as a marker of illness severity.^7^^,^^8^ Similar observations have been made in COVID-19, in which low LDL-C at admission was associated with disease severity and death,^9^^,^^10^ and during Extracorporeal Membrane Oxygenation (ECMO), in which lipoprotein concentrations are profoundly depressed.^11^

In the context of cardiac surgery and coronary revascularization, two large registries have suggested that patients with the lowest baseline LDL-C are at higher risk of death. Rezaee et al. reported excess mortality after revascularization in patients with a baseline LDL-C below 1.8 mmoL/L,^12^ and in the CREDO-Kyoto registry of 33,133 patients, four-year all-cause mortality rose from 9.2% in the highest LDL-C quintile to 19.4% in the lowest, with an adjusted hazard ratio of 1.29 (95% CI 1.16–1.44).^13^ In both studies, the authors attributed very low LDL-C to comorbidity burden rather than to a lipid mechanism, and in CREDO-Kyoto the excess mortality concerned cardiovascular and non-cardiovascular deaths alike.

The mechanisms underlying these associations remain poorly understood. Low lipoprotein concentrations may reflect an impaired general condition: in lung transplant candidates, a population in which chronic disease and undernutrition are prevalent, we previously found that a low pre-transplant HDL-C concentration was strongly associated with one-year mortality.^14^ Alternatively, low concentrations may reflect underlying inflammation, as in sepsis, COVID-19 or ECMO, in which lipoproteins are consumed and remodelled by the acute-phase response.^6^^,^^15^ HDL particles additionally carry anti-inflammatory, antioxidant and endotoxin-scavenging properties,^15^^,^^16^ and cardiac surgery with Cardiopulmonary Bypass (CPB) itself induces substantial changes in plasma lipoproteins.^17^^,^^18^ Whether a preoperative lipid profile, obtained before a discrete and standardised acute insult, carries prognostic information for early postoperative mortality has not, to our knowledge, been examined at scale in a predominantly non-coronary cardiac surgical population.

We therefore examined, in a large single-centre registry of consecutive patients undergoing cardiac surgery with CPB, whether preoperative LDL-C, TC, HDL-C and TG concentrations were associated with 28-day mortality after adjustment for measurable illness severity, and whether they carried prognostic information beyond EuroSCORE 2.^19^

## Materials and methods

### Study design

This is a retrospective analysis of a prospectively maintained institutional registry. Although the underlying registry enrolls patients prospectively and records in-hospital outcome, lipid values were merged with the clinical dataset after the fact, and the analytic cohort was defined by the retrospective availability of a lipid panel; we therefore describe the design as retrospective throughout. All consecutive patients who underwent cardiac surgery with CPB from 1 April 2013 to 31 December 2023 were eligible (the first and last operations in the analyzed cohort were performed on 12 April 2013 and 29 November 2023). Exclusion criteria were age under 18-years, surgery without CPB, absence of a preoperative lipid panel within 15-days before surgery, and reoperation within one year of a previous cardiac procedure. Patients with more remote prior cardiac surgery were eligible and included; redo surgery was entered as a covariate in the full-confounder sensitivity model.

### Data collection and ethics

This study was conducted through the merging of three databases. The first registry, Cardiobase, prospectively includes all adult patients who underwent cardiac surgery with CPB at our institution, together with their in-hospital outcome. The registry was approved on 19 March 2015 by the Comité d'Evaluation de l'Ethique des projets de Recherche Biomédicale (CEERB) Paris Nord (IRB 00006477; project 15-013), was declared to the Advisory Committee on Information Processing in Health Research (CCTIRS) and to the Commission Nationale de l'Informatique et des Libertés (CNIL, n° 1865399), and is registered at ClinicalTrials.gov (NCT03393169). As a non-interventional study based on routinely collected care data, it falls under the French regulatory framework in which written informed consent is not required: patients receive written information and are included unless they object. The approval letter and the registration record are provided as Supplementary Files 1a and 1b. The following data were collected in this registry: demographic information, medical history, type of surgery, perioperative details and in-hospital vital status.

Secondly, values for biological variables, including lipid concentrations, were retrieved from the biological database of our institution and merged with the patients' characteristics dataset. Finally, in-hospital vital status, recorded prospectively in the registry, was completed after discharge by linkage with the publicly available death records of the Institut National de la Statistique et des Études Économiques (INSEE) from 2013 to 2024,^20^ so that 28-day and 90-day mortality could be ascertained for patients discharged alive before these time points. These files record the date but not the cause of death.

### Analytical aspects

In the preoperative period for cardiac surgery, patients underwent routine check-ups including TC, TG, HDL-C and LDL-C. A maximum delay of 15-days between blood sampling and surgery was required; the sampling date of each panel was retrieved from the laboratory database and, when several panels were available within this window, the one closest to surgery was used. Analyses were performed in the Biochemistry Laboratory of Bichat-Claude Bernard Hospital. TC, HDL-C, LDL-C and TG concentrations were measured by routine enzymatic assays (Dimension VISTA® System, Siemens Healthineers™). The reference values were HDL-C > 1.00 (male) and > 1.20 (female) mmoL/L, TC 4.40–5.20 mmoL/L and TG 0.50–1.70 mmoL/L (Biochemistry Laboratory, Bichat-Claude Bernard Hospital). The laboratory changed its LDL-C method during the study period: until 2016 LDL-C was calculated by the Friedewald equation, and from 2017 onwards it was measured by a direct assay. Sixteen patients (0.8%) had no LDL-C value. Twelve were operated on before 2017 and thirteen had triglycerides above 3.9 mmoL/L, a concentration at which the Friedewald equation is not valid; the two counts overlap in twelve patients, so that all twelve missing values of the Friedewald era are explained by high triglycerides. The four patients without a value in the direct-assay era, one of them with high triglycerides, had no LDL-C result reported by the laboratory, for reasons not recorded. Fasting status is not recorded in the registry.

Non-HDL-C was derived as TC minus HDL-C.^21^ Apolipoprotein B is not measured routinely at our institution and could not be derived. Long-term statin use was defined as statin therapy recorded as part of the patient's usual medication at the preoperative anaesthesia consultation; the registry does not record the molecule, dose, duration of therapy or indication, nor the use of PCSK9 inhibitors. Markers of illness severity, nutrition and organ function (lymphocyte count, neutrophil-to-lymphocyte ratio, C-reactive protein, total protein, haemoglobin, platelet count, prothrombin time and creatinine) were also retrieved for sensitivity analyses.

### Objectives and outcomes

The primary comparison was the association between preoperative LDL-C concentration, analyzed as a continuous variable, and 28-day all-cause mortality. Associations with TC, HDL-C, TG and non-HDL-C were secondary. Ninety-day all-cause mortality was a secondary outcome. Analyses by type of surgery (isolated Coronary Artery Bypass Grafting [CABG] vs. other procedures), by preoperative status and by long-term statin use, and the quintile analyses, were exploratory and hypothesis-generating. A high-risk preoperative status was defined as the presence of infective endocarditis, urgent surgery or critical preoperative state, these conditions not being mutually exclusive; the remaining patients were classed as stable elective. To assess the influence of the preoperative condition on the lipid profile, four mutually exclusive groups were also compared: infective endocarditis, critical preoperative state, urgent procedure and stable elective patients, a patient meeting more than one criterion being classed in the first applicable group in that order. The statistical analysis plan was not registered in advance, and the primary comparison was designated during revision of this manuscript rather than before analysis.

### Statistical analysis

Continuous variables were expressed as medians with Interquartile Ranges (IQRs) and compared with the Mann-Whitney *U*-test. Categorical variables were expressed as counts and percentages and compared with Fisher's exact test or the Chi-Squared test, as appropriate. Comparisons across more than two groups used the Kruskal-Wallis test, and correlations between lipid parameters and markers of severity were assessed with Spearman's rank coefficient. For assessing changes over time, patients were grouped into two-year periods (2021–2023 forming the last period) and proportions were evaluated using the Cochran-Armitage test.^22^

Included and excluded patients were compared for all baseline variables using standardized mean differences in addition to p-values, since with more than 10,000 patients almost any difference reaches statistical significance; an absolute standardized mean difference above 0.10 was considered meaningful.

Each lipid parameter was modelled as a continuous variable using restricted cubic splines with three degrees of freedom in a logistic regression model. Departure from linearity was tested by likelihood ratio against the corresponding linear model, first without adjustment and then within the adjusted model, in which the overall contribution of the lipid parameter was also tested by likelihood ratio of the spline term against the model without the lipid parameter. Adjusted predicted probabilities were computed across the 1^st^ to 99^th^ percentile of each parameter. Quintile thresholds were used to divide the cohort into five subgroups of equal size for the display of absolute risks.

The multivariable adjustment set was prespecified from a directed acyclic graph representing the assumed causal structure (Supplementary Fig. S4), rather than derived from univariable screening. The minimal sufficient adjustment set for the total effect of preoperative cholesterol comprised proxies of illness severity and comorbidity together with statin use. EuroSCORE 2 was used as a composite severity score,^19^ since with 64 events the events-per-variable rule permits approximately six covariates. The final prespecified model therefore comprised the lipid parameter, EuroSCORE 2, long-term statin use, infective endocarditis, urgent surgery and critical preoperative state. CPB duration was deliberately excluded: it is measured after the exposure and, under the assumed structure, is a consequence of surgical complexity rather than a confounder of the cholesterol-mortality relationship, so that adjusting for it, adds nothing to the control of confounding while conditioning on a variable that may itself depend on the preoperative condition. Collinearity was assessed by the variance inflation factor, with a threshold of 5.^23^ Sensitivity analyses comprised multiple imputation of missing values, a full-confounder model additionally including heart failure, dialysis-dependent renal failure, redo surgery and cirrhosis, and a backward stepwise model based on the Akaike information criterion applied to the same candidate set; further sensitivity analyses addressed the interval between lipid sampling and surgery and the markers of illness severity, nutrition and organ function.

Missing values were not imputed in the primary analysis. Within the analysed cohort, no covariate of the prespecified model was missing and only 16 LDL-C values were; these were imputed by chained equations (20 imputations, 10 iterations) as a sensitivity analysis. The exposure was not imputed in the excluded patients. The influence of the interval between lipid sampling and surgery was examined by restricting the analysis to panels drawn within 7-, 3- and 1-day of surgery, by entering the interval as a covariate, and by testing its interaction with each lipid parameter.

Discrimination of each lipid parameter was assessed by the area under the Receiver Operating Characteristic (ROC) curve. The incremental value of each parameter beyond EuroSCORE 2 was assessed by the change in area under the ROC curve with DeLong's test,^24^ by likelihood ratio testing of nested models, by the integrated discrimination improvement, by the Brier score, and by calibration across deciles of predicted risk.

Differences between subgroups were assessed by formal likelihood ratio interaction testing rather than by comparing stratum-specific p-values: for each lipid parameter and each subgroup variable (type of surgery, preoperative status, statin use), a model with and a model without the product term between the two were compared, both adjusted for EuroSCORE 2, infective endocarditis, urgent surgery and critical preoperative state; the number of events is reported for every subgroup. The Benjamini–Hochberg procedure was applied to the primary family of lipid parameters, and Holm and Bonferroni corrections are also reported;^25^^,^^26^ no correction was applied to exploratory analyses, which are labelled as such.

All statistical analyses were performed using R software (R Core Team). A p-value below 0.05 was considered statistically significant. The study is reported in accordance with the STROCSS 2025 guideline for cohort studies in surgery (Appendix A)^27^ and with the STROBE statement for observational studies (Appendix B).^28^

## Results

### Cohort, selection and homogeneity

Between April 2013 and December 2023, 10,088 patients underwent cardiac surgery with CPB at Bichat-Claude Bernard Hospital. Sixty-nine were excluded for reoperation within one year of a previous cardiac procedure, two duplicate records were removed, and 8,031 patients were excluded because a complete preoperative lipid panel was not available, leaving 1,986 patients for analysis (Fig. 1). Vital status at 28-days was determined for all of them: 44 of the 64 deaths (69%) were already recorded in the registry and 20 (31%) were identified through the linkage with the national death records, which therefore contributed about a third of the events (60 and 28 of the 88 deaths at 90-days).

**Fig. 1:**
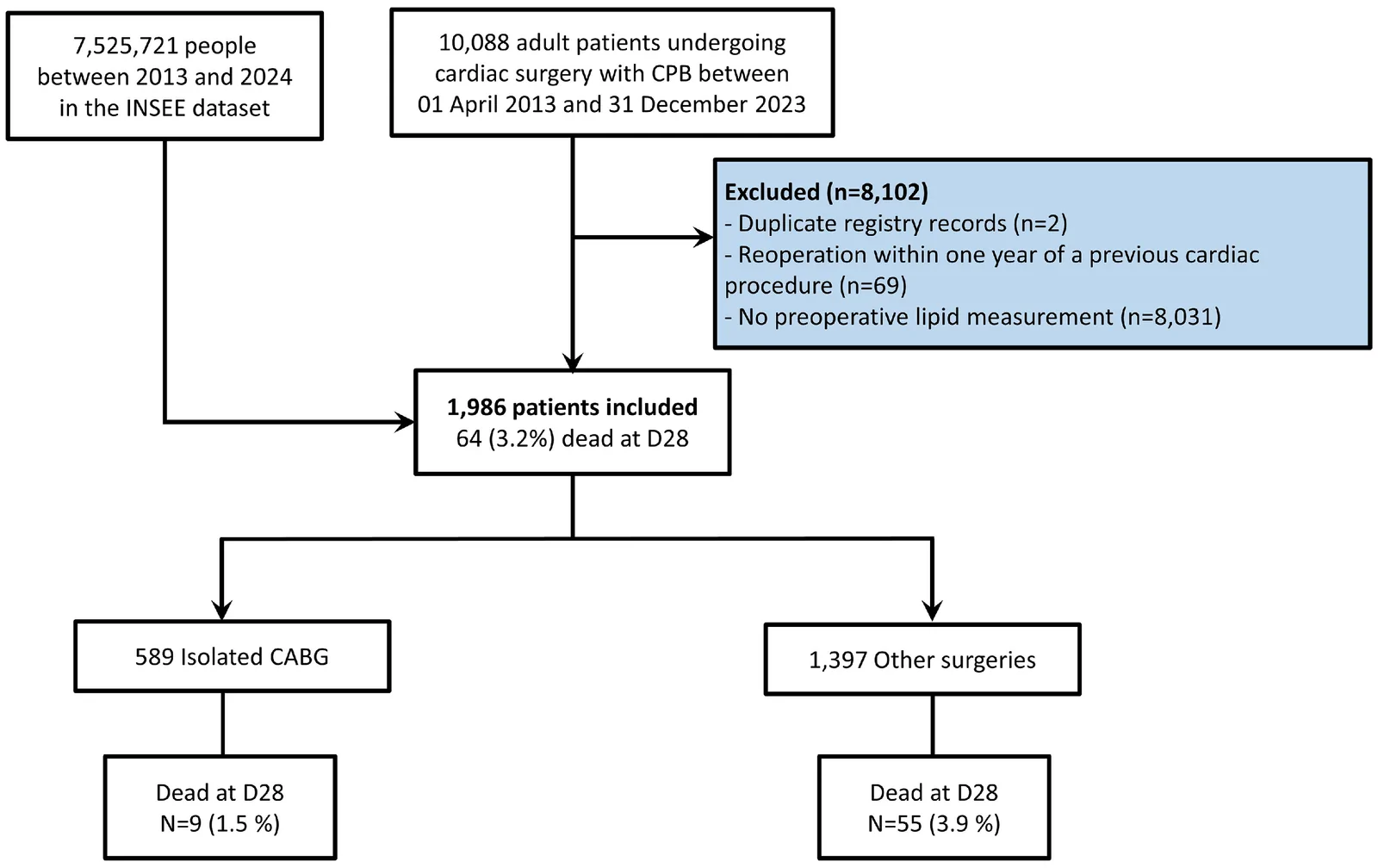
Flow chart. In-hospital vital status was recorded prospectively in the registry and completed after discharge by linkage with the INSEE national death records. Sixty-nine patients were excluded for reoperation within one year of a previous cardiac procedure and two duplicate records were removed; patients with more remote prior cardiac surgery were included. CABG, Coronary Artery Bypass Grafting; INSEE, Institut National de la Statistique et des Études Économiques.

Included and excluded patients differed in case mix rather than in severity (Supplementary Table S1). Included patients had less ischaemic heart disease and fewer of its correlates, namely statin prescription and isolated CABG, whereas valve surgery, heart failure, cardiac transplantation and longer cross-clamp and CPB durations were more frequent among them. Urgent surgery, infective endocarditis, critical preoperative state, EuroSCORE 2 and 28-day mortality were balanced between the two groups. Availability of the lipid panel rose from 10%–14% of operations in 2013–2016 to 22%‒31% from 2017 onwards, leading to uneven enrolment across the study period, with only 26% of participants recruited before 2017 (Supplementary Fig. S1A and S1G); this is consistent with progressive systematization of the preoperative work-up rather than with patient-level selection on severity. Twenty-eight-day mortality showed no significant trend over time (Supplementary Fig. S1B, Cochran-Armitage p = 0.128).

### Preoperative lipid concentrations

The lipid panel was drawn a median of 3 [1–7] days before surgery. This interval depended on the clinical context: urgent procedures and infective endocarditis were sampled closer to surgery, and the 82 patients (4.1%) sampled on the day of surgery combined the highest proportion of high-risk preoperative status (54%), the lowest median HDL-C (1.02 mmoL/L) and the highest 28-day mortality (7.3%) (Supplementary Table S2).

Median concentrations were 4.1 [3.3–5.0] mmoL/L for TC, 1.1 [0.9–1.4] mmoL/L for HDL-C, 2.4 [1.8–3.2] mmoL/L for LDL-C (available in 1,970 patients) and 1.4 [1.0–1.9] mmoL/L for TG. Median TG and HDL-C were within the reference range, whereas median TC was below it. The median LDL-C value was low in our cohort, although above the recommended target of 1.4 mmoL/L for patients at very high cardiovascular risk. TC did not vary across two-year periods, whereas TG, HDL-C and LDL-C differed between periods (Supplementary Fig. S1C–F); the rise in median LDL-C from 2.31 mmoL/L before 2017 to 2.50 mmoL/L thereafter coincides with the change of assay described in Methods (Analytical aspects). No age-related difference was observed for TC; however, HDL-C increased with age, whereas LDL-C showed the opposite trend (Supplementary Fig. S2). A clear sex-based difference emerged: female patients had higher TC, HDL-C and LDL-C and lower TG concentrations (Supplementary Fig. S3).

### Characteristics and postoperative course

The main comorbidities associated with 28-day mortality were chronic obstructive pulmonary disease, dialysis-dependent renal failure, cirrhosis and a left ventricular ejection fraction below 30%. Prior cardiac surgery, infective endocarditis, urgent surgery and a critical preoperative state were more frequent among patients who died within 28-days (Table 1).

**Table 1:** Baseline, preoperative and intraoperative characteristics according to 28-day mortality.

Characteristic	Overall (n = 1,986)	28-day survivors (n = 1,922)	28-day non-survivors (n = 64)	p-value
Demographics				
Age (years)	63 [52–71]	63 [52–71]	66 [57–73]	0.059
Male sex	1,349 (68)	1,309 (68)	40 (62)	0.343
Body mass index (kg/m²)	26 [23–30]	26 [24–30]	25 [22–28]	0.028
Obesity (BMI > 30 kg/m²)	501 (26)	490 (26)	11 (19)	0.286
Medical history				
Arterial hypertension	1,078 (54)	1,040 (54)	38 (59)	0.445
Diabetes mellitus	525 (26)	505 (26)	20 (31)	0.388
Dyslipidaemia	838 (42)	812 (42)	26 (41)	0.898
Active smoking	422 (21)	412 (21)	10 (16)	0.350
Peripheral arterial disease	184 (9.3)	174 (9.1)	10 (16)	0.080
Previous stroke	185 (9.3)	177 (9.2)	8 (12)	0.378
Ischaemic heart disease	798 (40)	775 (40)	23 (36)	0.519
Heart failure	206 (10)	195 (10)	11 (17)	0.091
LVEF < 30%	124 (6.2)	112 (5.8)	12 (19)	<0.001
Dialysis-dependent renal failure	43 (2.2)	38 (2.0)	5 (7.8)	0.011
COPD	96 (4.8)	88 (4.6)	8 (12)	0.010
Cirrhosis	23 (1.2)	19 (1.0)	4 (6.2)	0.005
Prior cardiac surgery	232 (12)	215 (11)	17 (27)	<0.001
Preoperative status				
Urgent surgery	153 (7.7)	139 (7.2)	14 (22)	<0.001
Infective endocarditis	109 (5.5)	96 (5.0)	13 (20)	<0.001
Critical preoperative state	95 (4.8)	83 (4.3)	12 (19)	<0.001
High-risk preoperative status^a^	270 (14)	242 (13)	28 (44)	<0.001
EuroSCORE 2 (%)	2.2 [1.2–4.2]	2.1 [1.2–4.0]	5.8 [2.5–15.9]	<0.001
Preoperative treatment				
Long-term statin	948 (48)	925 (48)	23 (36)	0.057
ACE inhibitor/ARB	920 (46)	894 (47)	26 (41)	0.375
Beta-blocker	1,124 (57)	1,093 (57)	31 (48)	0.200
Antiplatelet agent	917 (46)	895 (47)	22 (34)	0.057
Preoperative biology				
Haemoglobin (g/dL)	13.3 [11.7–14.5]	13.4 [11.8–14.5]	11.5 [10.2–12.7]	<0.001
Platelets (G/L)	221 [182–269]	221 [181–269]	224 [186–264]	0.929
Prothrombin time (%)	91 [81–100]	92 [81–100]	76 [61–90]	<0.001
Creatinine (µmoL/L)	86 [73–108]	86 [72–106]	118 [86–202]	<0.001
Type of surgery				
Isolated CABG	589 (30)	580 (30)	9 (14)	0.005
CABG and valve surgery	144 (7.3)	138 (7.2)	6 (9.4)	0.461
Isolated valve surgery	894 (45)	863 (45)	31 (48)	0.611
Thoracic aortic surgery, alone or combined	194 (9.8)	188 (9.8)	6 (9.4)	1.000
Cardiac transplantation	89 (4.5)	81 (4.2)	8 (12)	0.007
Other procedures^b^	76 (3.8)	72 (3.7)	4 (6.2)	0.306
Intraoperative data				
CPB duration (minutes)	75 [55–107]	74 [55–105]	126 [82–220]	<0.001
Aortic cross-clamp duration (minutes)	58 [43–81]	58 [43–80]	90 [55–140]	<0.001

Continuous variables are expressed as median [interquartile range] and compared with the Mann-Whitney *U*-test; categorical variables as n (%) and compared with Fisher's exact test.

a High-risk preoperative status: infective endocarditis, urgent surgery or critical preoperative state (not mutually exclusive). Infective endocarditis was defined as endocarditis treated with antibiotics at the time of surgery. The six types of surgery are mutually exclusive and total 1,986: thoracic aortic surgery includes procedures on the thoracic aorta performed alone or with either valve surgery or CABG, outside transplantation (patients with combined CABG, valve and aortic surgery are counted under CABG and valve surgery).

b Other procedures under CPB comprise resection of cardiac tumours, pericardiectomy and implantation of a left ventricular assist device, among others. Percentages are computed on non-missing values; values were missing for body mass index (n = 30), haemoglobin (38), platelets (55), prothrombin time (121), creatinine (39), CPB duration (46) and aortic cross-clamp duration (67), and complete for all other variables. ACE, Angiotensin-Converting Enzyme; ARB, Angiotensin Receptor Blocker; BMI, Body Mass Index; CABG, Coronary Artery Bypass Grafting; COPD, Chronic Obstructive Pulmonary Disease; CPB, Cardiopulmonary Bypass; EuroSCORE 2, European System for Cardiac Operative Risk Evaluation II; LVEF, Left Ventricular Ejection Fraction.

Among perioperative factors, a prolonged duration of CPB was associated with 28-day mortality (Table 1), as were higher post-CPB catecholamine requirements, prolonged mechanical ventilation and acute kidney injury (Table 2). Length of stay in the intensive care unit was longer among 28-day non-survivors than among survivors, whereas hospital length of stay was shorter, as expected, since death terminates the stay; hospital length of stay in this group should not be interpreted as a measure of severity.

**Table 2:** Postoperative course and outcomes according to 28-day mortality.

Variable	Overall (n = 1,986)	28-day survivors (n = 1,922)	28-day non-survivors (n = 64)	Missing, n	p-value
Post-CPB catecholamine support	1,223 (62)	1,169 (61)	54 (84)	0	<0.001
Catecholamine support in ICU	1,137 (67)	1,093 (66)	44 (77)	281	0.115
Duration of mechanical ventilation (hours)	6 [5–9]	6 [5–8]	96 [7–240]	335	<0.001
Mechanical ventilation > 48h	175 (11)	150 (9.3)	25 (61)	335	<0.001
Acute kidney injury	38 (1.9)	31 (1.6)	7 (11)	0	<0.001
Renal replacement therapy	17 (1.2)	15 (1.0)	2 (9.1)	516	0.025
Surgical re-intervention	78 (3.9)	76 (4.0)	2 (3.1)	0	1.000
ICU length of stay (days)^a^	4.0 [2.0–7.0]	4.0 [2.0–6.0]	6.0 [3.0–13.8]	306	0.017
Hospital length of stay (days)^a^	14.0 [10.0–21.0]	14.0 [10.0–21.0]	7.5 [3.2–15.0]	432	<0.001

Continuous variables are expressed as median [interquartile range] and compared with the Mann-Whitney *U*-test; categorical variables as n (%) and compared with Fisher's exact test. Percentages are computed on non-missing values; the number of missing values is given for each variable. The duration of mechanical ventilation is not recorded in 23 of the 64 non-survivors, so that the proportion ventilated beyond 48-hours in this group is computed on 41 patients. Acute kidney injury and renal replacement therapy are binary registry items without severity staging; renal replacement therapy was not recorded in most patients operated on from 2020 onwards.

a ICU length of stay computed from the dates of ICU admission and discharge; hospital length of stay from the date of surgery to the date of hospital discharge. CPB, Cardiopulmonary Bypass; ICU, Intensive Care Unit.

### Low preoperative cholesterol was associated with 28-day mortality, and the relationship was not linear

Patients who died within 28-days had lower TC (3.65 vs. 4.13 mmoL/L, p = 0.001), HDL-C (1.02 vs. 1.14 mmoL/L, p = 0.004) and LDL-C (2.10 vs. 2.46 mmoL/L, p = 0.010) concentrations than survivors, whereas TG did not differ significantly (p = 0.059) (Fig. 2).

**Fig. 2:**
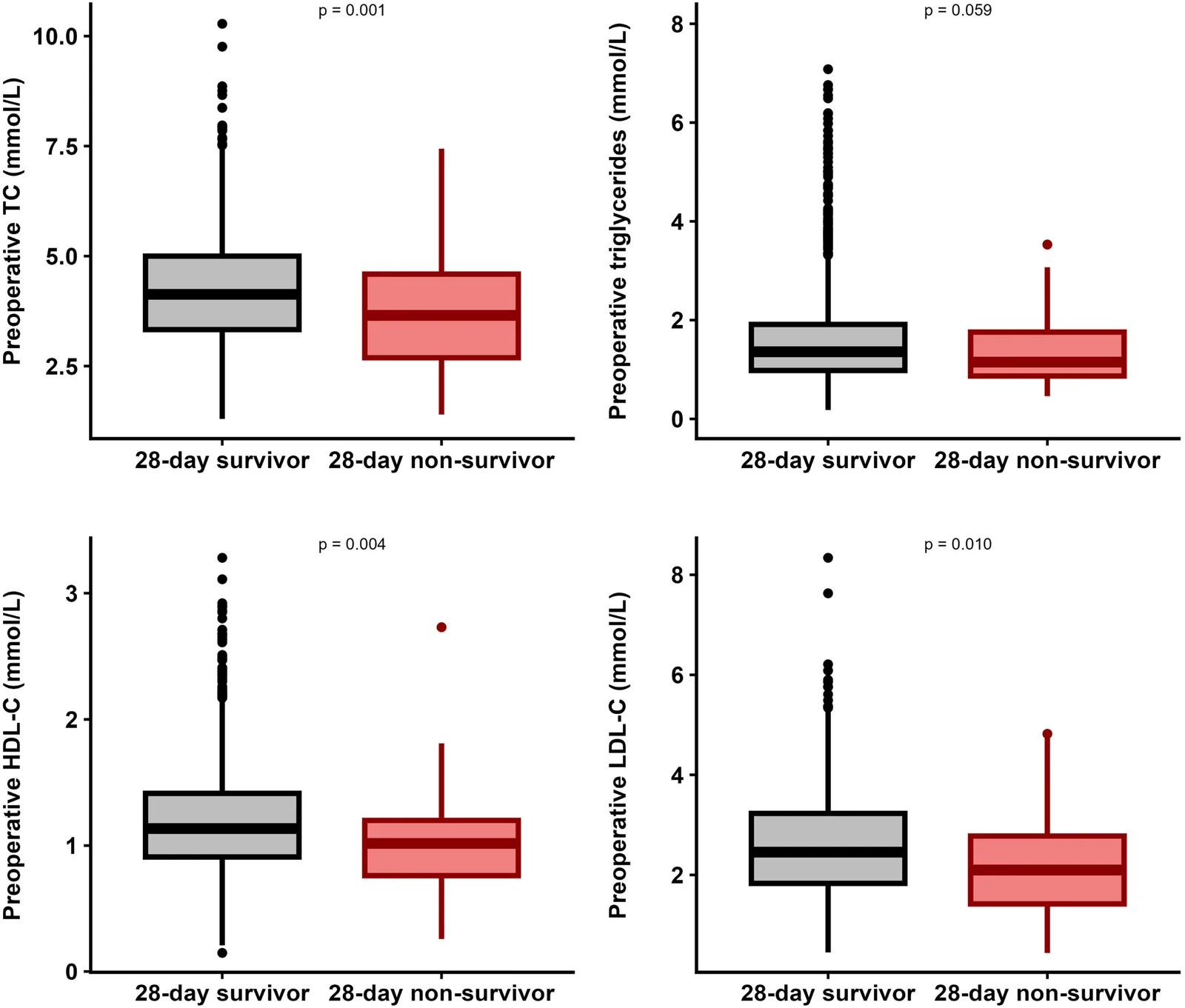
**Preoperative lipid concentrations according to 28-day mortality.** Total Cholesterol (TC), Triglycerides (TG), High-Density Lipoprotein Cholesterol (HDL-C) and Low-Density Lipoprotein Cholesterol (LDL-C) in survivors (n = 1,922) and 28-day non-survivors (n = 64). Boxes show the median and interquartile range; p-values from the Mann-Whitney *U*-test. The triglyceride axis is truncated at 8 mmoL/L.

This relationship was not linear. Absolute 28-day mortality across LDL-C quintiles was 5.8%, 2.8%, 3.3%, 1.3% and 3.0% from the lowest to the highest quintile (p = 0.010), the upper boundary of the lowest quintile being 1.66 mmoL/L (Fig. 3). Modelled with restricted cubic splines and without adjustment, the relationship with 28-day mortality departed significantly from linearity for TC (p = 0.029) and for LDL-C (p = 0.047), with predicted mortality rising steeply below approximately 3 mmoL/L and 2 mmoL/L respectively, but not for HDL-C (p = 0.27) or TG (p = 0.81); the curves kept the same shape after adjustment for the prespecified set (Fig. 4).

**Fig. 3:**
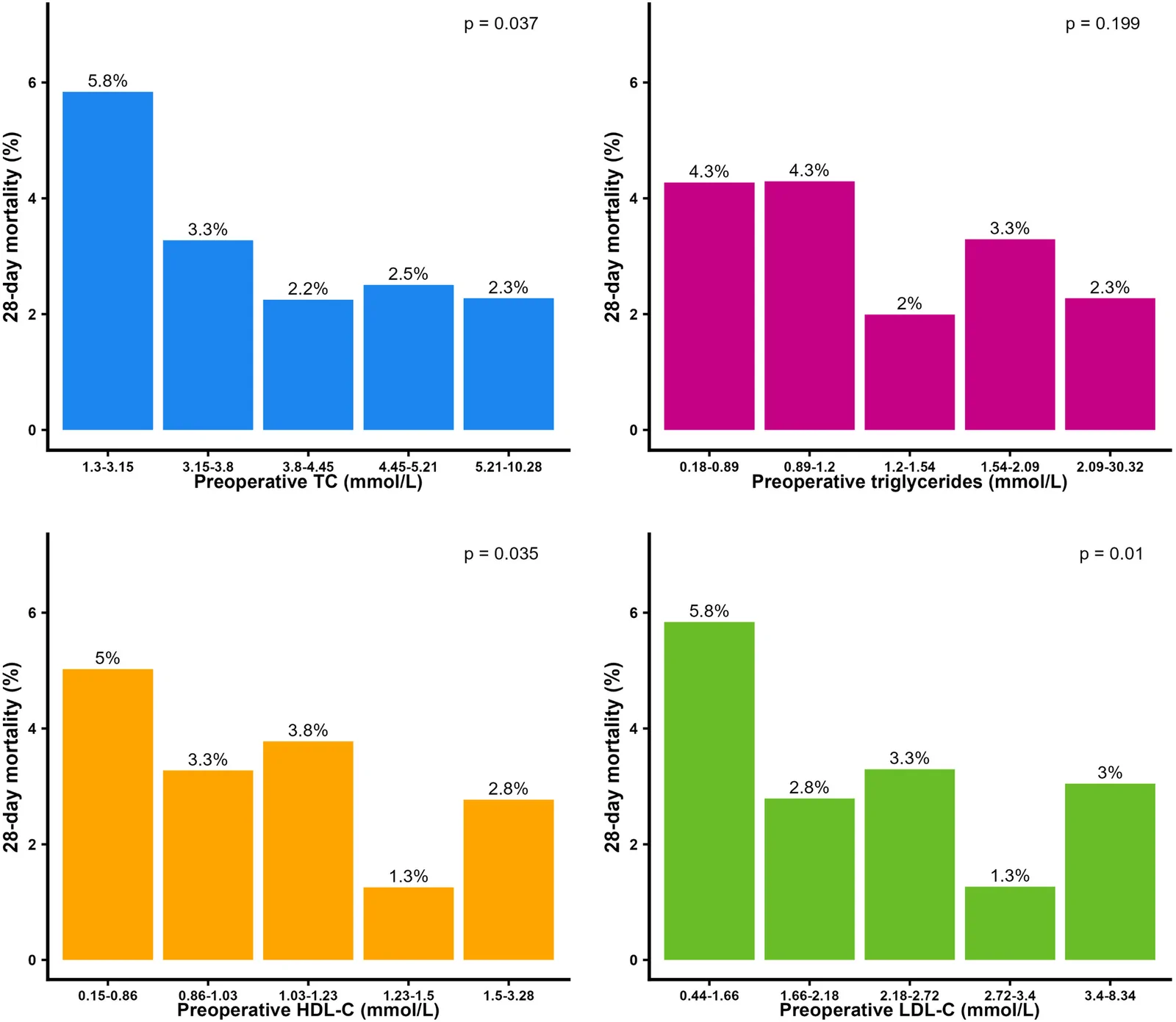
**Absolute 28-day mortality by quintile of preoperative TC, TG, HDL-C and LDL-C.** Quintile boundaries (mmoL/L) are given under each bar; p-values from Fisher's exact test across quintiles. These analyses are exploratory.

**Fig. 4:**
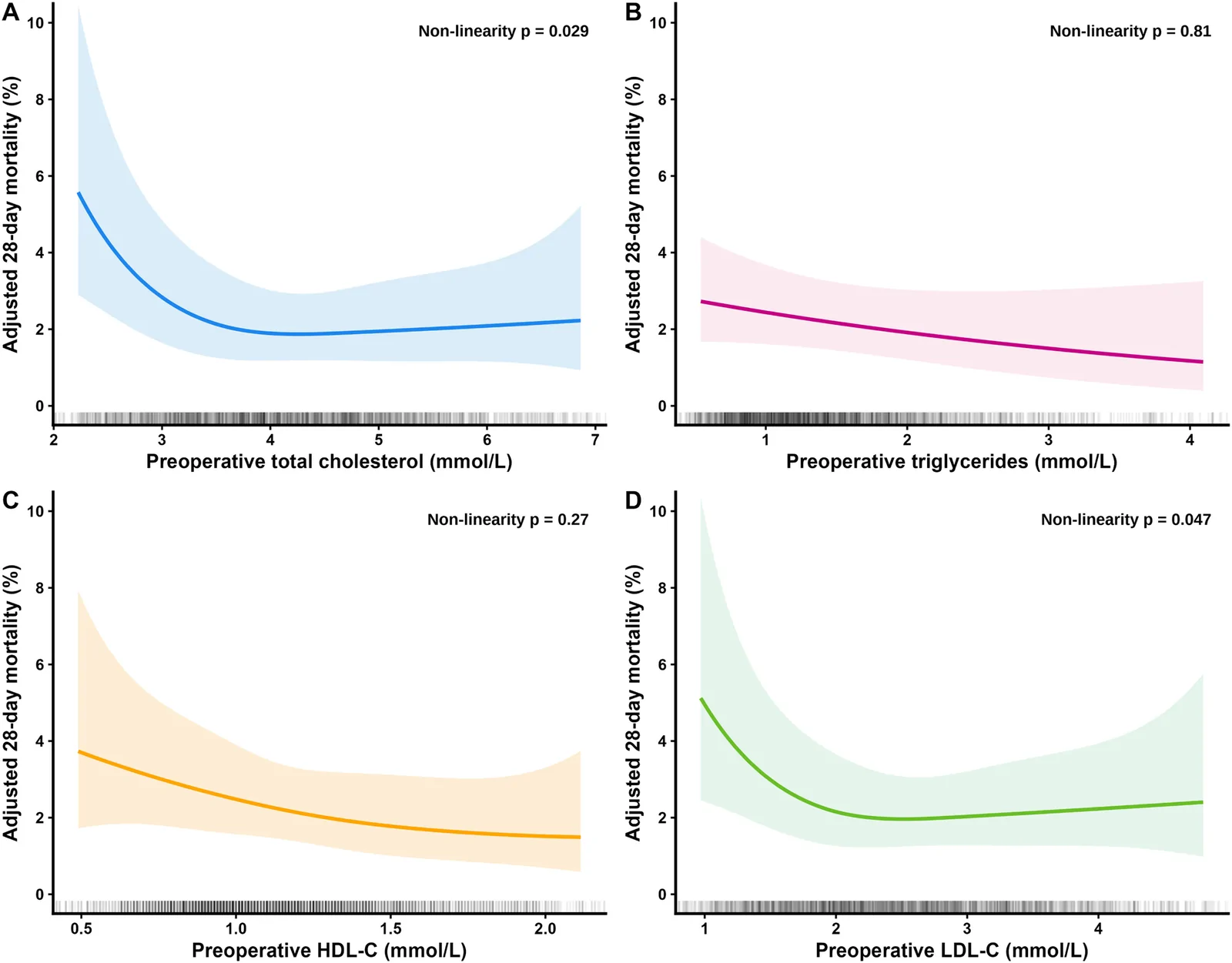
**Adjusted 28-day mortality across the range of preoperative TC (A), TG (B), HDL-C (C) and LDL-C (D).** Each parameter was modelled with restricted cubic splines (3 degrees of freedom) in a logistic model adjusted for EuroSCORE 2, long-term statin use, infective endocarditis, urgent surgery and critical preoperative state, with covariates set at the median (EuroSCORE 2) or at zero. Shaded areas are 95% confidence intervals; the rug shows the distribution of observed values. Axes are truncated at the 1^st^ and 99^th^ percentiles. The p-value on each panel tests departure from linearity by likelihood ratio in the unadjusted spline model; the corresponding tests in the adjusted model are given in Results.

### The association did not persist after adjustment

Adjustment was performed for the set prespecified from the directed acyclic graph (Supplementary Fig. S4). Adjusted odds ratios per 1 mmoL/L increase were 0.777 (0.607–0.994, p = 0.045) for TC, 0.780 (0.546–1.115, p = 0.173) for TG, 0.538 (0.261–1.107, p = 0.092) for HDL-C and 0.803 (0.595–1.084, p = 0.152) for LDL-C; after Benjamini–Hochberg correction across the primary family, no parameter remained associated with 28-day mortality (all *q* = 0.173; Table 3, Supplementary Table S3). For each lipid parameter, EuroSCORE 2 was the only covariate associated with mortality (Table 4). Non-HDL-C behaved indistinguishably from LDL-C (adjusted odds ratio = 0.812, 95% CI 0.623–1.058) and is not reported separately in the tables. The same adjustment also removed the non-linearity: in the adjusted spline model the departure from linearity was no longer significant (TC p = 0.061, LDL-C p = 0.142), and a global three-degree-of-freedom test of the spline term against the model without the lipid gave p = 0.020 for TC and p = 0.109 for LDL-C. The absence of an adjusted association is therefore not an artefact of imposing a linear term on a non-linear relationship.

**Table 3:** Unadjusted and adjusted odds ratios for 28-day mortality per 1 mmoL/L increase in each preoperative lipid parameter.

Lipid parameter	n	Unadjusted OR (95% CI)	p	Adjusted OR (95% CI)	p	*q* (BH)
Total cholesterol	1,986	0.688 (0.548–0.863)	0.001	0.777 (0.607–0.994)	0.045	0.173
Triglycerides	1,986	0.715 (0.503–1.016)	0.061	0.780 (0.546–1.115)	0.173	0.173
HDL-C	1,986	0.361 (0.181–0.721)	0.004	0.538 (0.261–1.107)	0.092	0.173
LDL-C	1,970	0.703 (0.533–0.927)	0.012	0.803 (0.595–1.084)	0.152	0.173

Each lipid parameter was entered separately to avoid collinearity (variance inflation factors all < 2). The adjustment set was prespecified from the directed acyclic graph shown in Supplementary Fig. S4 and comprises EuroSCORE 2, long-term statin use, infective endocarditis, urgent surgery and critical preoperative state; CPB duration, a post-exposure variable, was not included. The full models are given in Table 4. *q*-values are Benjamini-Hochberg corrected across the four lipid parameters of the primary family; Holm and Bonferroni corrections are given in Supplementary Table S3. The *q*-values are identical across the family because the step-up procedure enforces monotonicity, the least significant comparison (TG, p = 0.173) bounding all others.

BH, Benjamini-Hochberg; CI, Confidence Interval; CPB, Cardiopulmonary Bypass; EuroSCORE 2, European System for Cardiac Operative Risk Evaluation II; HDL-C, High-Density Lipoprotein Cholesterol; LDL-C, Low-Density Lipoprotein Cholesterol; OR, Odds Ratio.

**Table 4:** Prespecified multivariable models for 28-day mortality: adjusted odds ratios (95% CI) for every covariate, one model per lipid parameter.

Covariate	TC	TG	HDL-C	LDL-C
Lipid parameter (per 1 mmoL/L)	0.777 (0.607–0.994) p = 0.045	0.780 (0.546–1.115) p = 0.173	0.538 (0.261–1.107) p = 0.092	0.803 (0.595–1.084) p = 0.152
EuroSCORE 2 (per point)	1.089 (1.058–1.120) p < 0.001	1.086 (1.055–1.118) p < 0.001	1.093 (1.061–1.125) p < 0.001	1.087 (1.056–1.119) p < 0.001
Long-term statin therapy	0.727 (0.409–1.290) p = 0.276	0.858 (0.490–1.502) p = 0.592	0.788 (0.450–1.381) p = 0.405	0.736 (0.411–1.316) p = 0.301
Infective endocarditis	1.671 (0.748–3.732) p = 0.210	2.135 (0.977–4.666) p = 0.057	1.571 (0.678–3.637) p = 0.292	1.841 (0.829–4.089) p = 0.134
Urgent surgery	1.538 (0.673–3.511) p = 0.307	1.720 (0.765–3.864) p = 0.189	1.600 (0.705–3.627) p = 0.261	1.591 (0.698–3.623) p = 0.269
Critical preoperative state	0.539 (0.185–1.572) p = 0.258	0.651 (0.227–1.873) p = 0.426	0.503 (0.169–1.498) p = 0.217	0.602 (0.209–1.735) p = 0.347
n	1,986	1,986	1,986	1,970

One logistic model per lipid parameter, each comprising the lipid parameter, EuroSCORE 2, long-term statin use, infective endocarditis, urgent surgery and critical preoperative state. EuroSCORE 2 is a composite score that already contains age, sex, renal function, left ventricular ejection fraction and procedural complexity, which is why these are not entered separately; infective endocarditis, urgent surgery and critical preoperative state are retained as separate terms because they are the measured components of the acute preoperative illness that, under the assumed causal structure, both depresses cholesterol and increases mortality, and EuroSCORE 2 captures them only partly; this comes at the cost of partial double counting. The odds ratio below 1 for critical preoperative state should be read in that light and not as a protective effect. Variance inflation factors were all below 2. CI, Confidence Interval; HDL-C, High-Density Lipoprotein Cholesterol; LDL-C, Low-Density Lipoprotein Cholesterol; TC, Total Cholesterol; TG, Triglycerides.

The estimates were unchanged when the analysis was restricted to panels drawn within 7- or 3-days of surgery (Supplementary Table S4), after multiple imputation of the missing LDL-C values (Supplementary Table S5, Supplementary Fig. S5), and in a full-confounder model further adjusted for heart failure, dialysis-dependent renal failure, redo surgery and cirrhosis (Supplementary Table S6). They were also similar in the two LDL-C assay eras (adjusted odds ratio for LDL-C 0.597, 95% CI 0.343–1.040, before 2017 and 0.923, 0.640–1.332, from 2017; interaction p = 0.18) and unchanged when calendar year was entered as a covariate (0.808, 0.599–1.091). In a backward stepwise model, the lipid parameter was retained for TC, TG and HDL-C with unchanged estimates and dropped for LDL-C (Supplementary Table S7).

### Lipid concentrations added no information beyond EuroSCORE 2

Discrimination of individual lipid parameters was poor, with areas under the ROC curve between 0.567 and 0.619 (Supplementary Fig. S6A). Given this, no threshold is proposed.

EuroSCORE 2 alone achieved an area under the curve of 0.740. Adding any lipid parameter changed this by between 0.010 and 0.025, and none of these changes was significant (DeLong p ≥ 0.35) (Table 5, Supplementary Fig. S6B). The integrated discrimination improvement ranged from +0.0002 to +0.0043 and the Brier score was unchanged to the third decimal. Likelihood ratio testing was nominally significant for TC (p = 0.029) and HDL-C (p = 0.037), the expected pattern of a non-zero regression coefficient without gains in discrimination. Calibration of the models with and without LDL-C was comparable (Supplementary Fig. S7). Preoperative lipid concentrations therefore add no measurable information to established preoperative risk assessment, although with 64 events this analysis could not have detected a small one.

**Table 5:** Discrimination and incremental value of preoperative lipid parameters over EuroSCORE 2.

Model	AUC	ΔAUC	p (DeLong)	p (LRT)	IDI	Brier score
EuroSCORE 2 alone	0.740	‒	‒	‒	‒	0.0295
+ Total cholesterol	0.764	+0.025	0.45	0.029	+0.0015	0.0294
+ Triglycerides	0.752	+0.012	0.56	0.179	+0.0003	0.0295
+ HDL-C	0.749	+0.010	0.72	0.037	+0.0043	0.0293
+ LDL-C	0.764	+0.024	0.35	0.127	+0.0002	0.0295

All models were fitted in the 1,970 patients with a complete lipid panel including LDL-C (64 deaths), so that the areas under the curve are comparable across rows. Individual lipid parameters alone had areas under the curve of 0.619 (TC), 0.567 (TG), 0.607 (HDL-C) and 0.595 (LDL-C). AUC, Area Under the receiver operating Characteristic Curve; IDI, Integrated Discrimination Improvement; LRT, Likelihood Ratio Test.

### Lipid concentrations differed according to the type of preoperative illness

To assess the influence of the preoperative condition on the lipid profile, four groups in which this condition was expected to differ were compared: patients with infective endocarditis, patients in a critical preoperative state, patients undergoing an urgent procedure and stable elective patients (see Objectives and outcomes); median EuroSCORE 2 ranged from 1.95 in stable elective patients to 12.71 in critical preoperative state. Median HDL-C differed across the four groups (Kruskal-Wallis p < 0.001), being highest in stable elective patients (1.17 mmoL/L) and lowest in infective endocarditis (0.74 mmoL/L), with 0.99 mmoL/L in urgent procedures and 0.90 mmoL/L in critical preoperative state; 28-day mortality was 2.1%, 11.9%, 8.9% and 9.8% respectively (Supplementary Table S8, Supplementary Fig. S8). TC and LDL-C also differed across the four groups (both p < 0.001), their lowest medians being observed in the critical preoperative state group rather than in endocarditis. The four groups are not ordered by severity, since EuroSCORE 2 is highest in the critical preoperative state group and intermediate in endocarditis (4.19), and they are not ordered by clinical acuity either; the differences therefore track the type of acute illness rather than a single severity axis.

Among the markers of inflammation, organ function and nutrition available in this cohort, the most informative correlation was that between HDL-C and C-reactive protein (Spearman ρ = -0.251, p < 0.001); the remaining correlations were weak (all |ρ| < 0.24) and are reported in Supplementary Table S9. C-reactive protein and albumin are not part of the routine preoperative work-up at our institution: C-reactive protein was available in 1,162 patients (58.5%) and preferentially in patients following the elective pathway (Supplementary Table S10), so that a model adjusted for it would exclude the acutely ill patients who carry the association; such a model is reported as a sensitivity analysis only. In the 1,159 of these patients who also had a lymphocyte count (35 deaths), the odds ratio for HDL-C was 0.424 (0.155–1.160) in the prespecified model and 0.358 (0.131–0.978, p = 0.045) after adding C-reactive protein and lymphocyte count, whereas TC and LDL-C remained null; in these models the lymphocyte count, not C-reactive protein, was associated with mortality (LDL-C model: 0.341 per G/L, 95% CI 0.180–0.645, p < 0.001, versus 1.061 per 10 mg/L, 0.982–1.147, p = 0.134). With 35 deaths and a subgroup selected on the elective pathway, we do not interpret the isolated HDL-C result (Supplementary Table S11).

In the subgroup with available lymphocyte count, haemoglobin and prothrombin time (1,215 patients for TC and HDL-C, 1,201 for LDL-C, 34 deaths), the odds ratios were already null under the prespecified model alone (0.894 for TC, 0.966 for LDL-C) and were not further changed by adding these markers (1.123 and 1.212 respectively) (Table 6). In Model 2, prothrombin time was associated with mortality (LDL-C model: 0.949 per %, 95% CI 0.928–0.970, p < 0.001) whereas lymphocyte count (0.559 per G/L, p = 0.080) and hemoglobin (0.885 per g/dL, p = 0.254) were not. The association is therefore lost on restriction to this subgroup rather than by adjustment for the markers, and with 34 deaths these estimates are unstable.

**Table 6:** Adjusted odds ratios (95% CI) for 28-day mortality for every covariate, before (Model 1) and after (Model 2) additional adjustment for lymphocyte count, haemoglobin and prothrombin time, one model per lipid parameter.

Covariate	TC		HDL-C		LDL-C	
	Model 1	Model 2	Model 1	Model 2	Model 1	Model 2
Lipid parameter (per 1 mmoL/L)	0.894 (0.644–1.243) p = 0.506	1.123 (0.816–1.546) p = 0.475	0.434 (0.155–1.216) p = 0.112	0.629 (0.226–1.752) p = 0.375	0.966 (0.654–1.429) p = 0.864	1.212 (0.824–1.783) p = 0.329
EuroSCORE 2 (per point)	**1.101 (1.057–1.146) p < 0.001**	**1.095 (1.052–1.140) p < 0.001**	**1.109 (1.064–1.156) p < 0.001**	**1.097 (1.054–1.143) p < 0.001**	**1.101 (1.057–1.147) p < 0.001**	**1.096 (1.053–1.141) p < 0.001**
Long-term statin therapy	0.775 (0.358–1.677) p = 0.517	0.985 (0.449–2.157) p = 0.969	0.780 (0.364–1.675) p = 0.525	0.949 (0.433–2.082) p = 0.896	0.800 (0.367–1.746) p = 0.576	1.022 (0.464–2.252) p = 0.957
Infective endocarditis	2.360 (0.774–7.195) p = 0.131	1.499 (0.503–4.464) p = 0.467	1.722 (0.521–5.696) p = 0.373	1.191 (0.372–3.808) p = 0.768	2.550 (0.842–7.728) p = 0.098	1.493 (0.503–4.430) p = 0.470
Urgent surgery	0.701 (0.173–2.832) p = 0.618	0.460 (0.121–1.750) p = 0.255	0.681 (0.168–2.768) p = 0.592	0.436 (0.116–1.641) p = 0.219	0.732 (0.183–2.929) p = 0.659	0.473 (0.126–1.779) p = 0.268
Critical preoperative state	0.653 (0.144–2.952) p = 0.580	0.419 (0.102–1.725) p = 0.228	0.519 (0.111–2.422) p = 0.404	0.365 (0.087–1.534) p = 0.169	0.695 (0.155–3.113) p = 0.634	0.420 (0.103–1.713) p = 0.226
Lymphocyte count (per G/L)	‒	0.562 (0.293–1.079) p = 0.083	‒	0.544 (0.282–1.051) p = 0.070	‒	0.559 (0.291–1.072) p = 0.080
Haemoglobin (per g/dL)	‒	0.883 (0.718–1.087) p = 0.241	‒	0.899 (0.731–1.106) p = 0.313	‒	0.885 (0.717–1.092) p = 0.254
Prothrombin time (per %)	‒	**0.949 (0.928–0.971) p < 0.001**	‒	**0.953 (0.932–0.974) p < 0.001**	‒	**0.949 (0.928–0.970) p < 0.001**
n / deaths	1,215 / 34	1,215 / 34	1,215 / 34	1,215 / 34	1,201 / 34	1,201 / 34

Fitted in the subgroup with available lymphocyte count, hemoglobin and prothrombin time. Model 1 is the prespecified model refitted in this subgroup; Model 2 adds the three markers. Values in bold: p < 0.05. CI, Confidence Interval; HDL-C, High-Density Lipoprotein Cholesterol; LDL-C, Low-Density Lipoprotein Cholesterol; TC, Total Cholesterol.

### Subgroup and statin analyses

The number of deaths was 9 of 589 in isolated CABG and 55 of 1,397 in other procedures, 28 of 270 in patients with a high-risk preoperative status (infective endocarditis, urgent surgery or critical preoperative state) and 36 of 1,716 stable elective patients, and 23 of 948 in statin-treated patients and 41 of 1,038 in the others (Supplementary Fig. S9). Formal likelihood ratio interaction testing between each lipid parameter and type of surgery, preoperative status and statin use was nominally significant for LDL-C and TC with type of surgery (p = 0.012 and 0.044) and with statin use (p = 0.013 and 0.016), but no interaction survived Benjamini–Hochberg correction across the twelve tests (all *q* ≥ 0.062), and none was found with preoperative status (p = 0.29–0.79). With nine deaths in the isolated CABG stratum, these analyses are exploratory and support no conclusion in any individual subgroup.

Long-term statin therapy was recorded in 47.7% of patients. Patients on statins had lower TC (3.86 vs. 4.43 mmoL/L), LDL-C (2.14 vs. 2.74 mmoL/L) and HDL-C (1.10 vs. 1.17 mmol/L) concentrations than those not on statins, and higher TG (Fig. 5). Since statin therapy lowers the exposure and is itself a marker of cardiovascular disease burden, it was included in the prespecified adjustment set; its interaction with LDL-C did not survive correction for multiplicity, and no inference about statin therapy is drawn from these data.

**Fig. 5:**
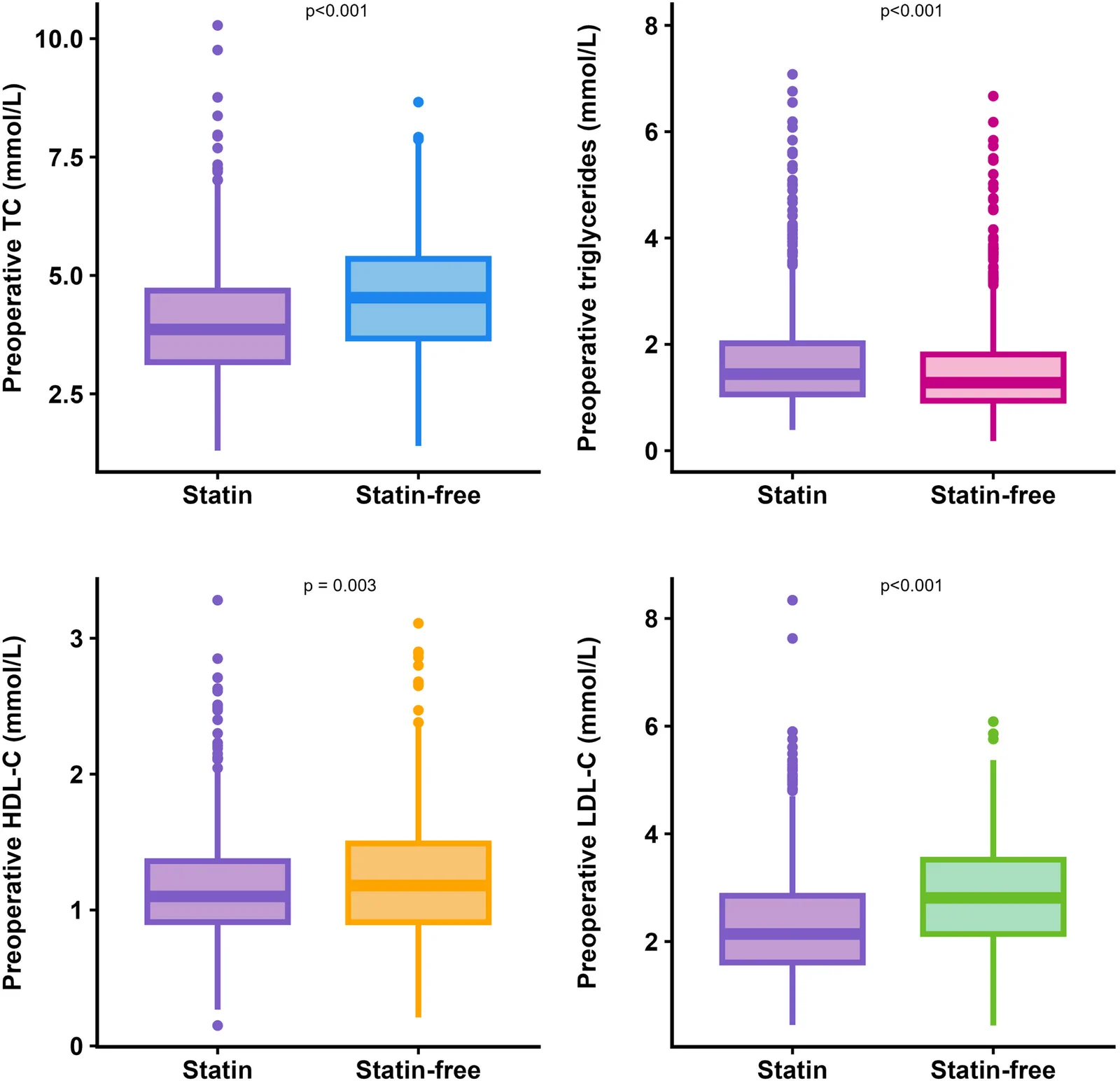
**Preoperative TC, TG, HDL-C and LDL-C concentrations according to long-term statin therapy.** Boxes show the median and interquartile range; p-values from the Mann-Whitney *U*-test. The triglyceride axis is truncated at 8 mmoL/L.

Two subgroups deserve separate mention because they isolate the exposure from the conditions that are thought to depress it. Among the 1,716 stable elective patients (36 deaths), in whom the lipid panel was drawn before any acute insult, the odds ratios adjusted for EuroSCORE 2 and statin use (the three acute-illness terms of the prespecified set being null by construction) were 0.725 (0.528–0.996, p = 0.047) for TC and 0.743 (0.503–1.098, p = 0.137) for LDL-C. Among the 1,038 patients not receiving a statin (41 deaths), in whom the exposure is not modified by treatment, no association was found for any parameter after adjustment for the remaining terms of the prespecified set (LDL-C 1.024, 0.711–1.475, p = 0.900; TC 0.937, 0.697–1.259, p = 0.666).

### Ninety-day mortality

Eighty-eight patients (4.4%) had died at 90-days. The adjusted estimates at 90-days were consistent with those at 28-days for every parameter (LDL-C 0.811, 95% CI 0.627–1.049, p = 0.110; Supplementary Table S12). Total cholesterol was the only parameter nominally associated with 90-day mortality (0.799 per 1 mmoL/L, 0.649–0.984, p = 0.035), as it was at 28-days (Table 3); neither association survived correction for multiplicity across the primary family (Benjamini-Hochberg *q* = 0.140 at 90-days and 0.173 at 28-days).

## Discussion

The main finding of this study of 1,986 patients is that low preoperative TC, HDL-C and LDL-C concentrations were associated with 28-day mortality after cardiac surgery with CPB in unadjusted analysis, but that LDL-C, the primary comparison, was no longer associated with mortality after adjustment for markers of illness severity, that only TC retained a borderline association which did not survive correction for multiplicity, that discrimination was poor, and that lipid concentrations added no information to EuroSCORE 2. Taken together with the low HDL-C observed in patients with an acute preoperative illness, these results suggest that a low preoperative cholesterol concentration is a marker of underlying illness severity rather than a causal determinant of postoperative death.

### Positioning within the existing literature

Our interpretation is concordant with that of the two revascularization registries that motivated this work, those of Rezaee et al.^12^ and CREDO-Kyoto.^13^ In the latter, low LDL-C clustered with anaemia, thrombocytopenia, dialysis-dependent renal failure, cirrhosis and malignancy, and the excess mortality was present for cardiovascular and non-cardiovascular causes alike, which a cholesterol-specific mechanism does not readily explain. The same pattern is described in sepsis^8^ and in COVID-19.^9^^,^^10^ It is also consistent with the absence of a mortality benefit of LDL-C lowering when baseline LDL-C is already below 2.6 mmoL/L:^5^ a low LDL-C identifies patients in whom further lowering is unlikely to help and, in the acute setting, patients whose prognosis is driven by something other than cholesterol. Three features distinguish the present work from those registries: the outcome is early, 28-day perioperative mortality rather than late mortality; the population is not restricted to coronary disease, 70.3% of patients undergoing procedures other than isolated CABG; and every patient is exposed to a discrete, scheduled acute insult. It reaches a different result: in CREDO-Kyoto the excess mortality survived adjustment, whereas here it did not, although with 64 events our confidence interval does not exclude an effect of the size they report.

### Low cholesterol as a marker of illness severity

The fall in TC, HDL-C and LDL-C during the acute-phase response is proportionate to the severity of the underlying illness.^6^^,^^8^ Our data are consistent with this at several levels. HDL-C was lowest in patients with infective endocarditis and correlated inversely with C-reactive protein. The association with mortality did not survive adjustment for the prespecified severity set. After adjustment, triglycerides, which are not part of the cholesterol pool and are not associated with outcome in sepsis, behaved no differently from LDL-C (odds ratios 0.780 and 0.803), although the width of the confidence intervals limits the weight of this observation. This interpretation rests on the convergence of these indirect indicators; nutritional status itself was not measured, and the data do not allow undernutrition and inflammation to be separated as the source of low lipoprotein concentrations.

Read this way, the present results also sit consistently alongside two of our earlier cohorts that at first sight disagree. Under ECMO, where lipoproteins were measured after cannulation in patients uniformly in extreme critical illness, HDL-C was profoundly and near-universally depressed and carried no prognostic information;[11] in lung transplant candidates, sampled before the insult in patients who were for the most part clinically stable, a low pre-transplant HDL-C was the variable most strongly associated with one-year mortality.^14^ Cholesterol concentration appears to carry prognostic information only until the acute insult has collapsed it, and the present cohort contains both situations within a single population, from stable elective patients to patients with infective endocarditis (Supplementary Table S8).

Two limitations of this reasoning must be acknowledged. First, no adjustment set can fully resolve reverse causation when illness severity is measured with error, and EuroSCORE 2, however comprehensive, is an imperfect proxy. Second, the design, a single preoperative measurement merged retrospectively, cannot in principle distinguish marker from cause. This is precisely why we present the finding as an association with a marker of severity and make no causal claim.

One qualification must be made to this interpretation. In the prespecified model, total cholesterol is the only parameter that keeps a nominal association with mortality after adjustment, at 28-days (p = 0.045) and at 90-days (p = 0.035), and the only one for which the adjusted spline model shows an overall effect (p = 0.020); the association also persists, at the same borderline level, in the 1,716 stable elective patients in whom the panel was drawn before any acute insult (0.725, 0.528–0.996, p = 0.047). None of these results survives correction for multiplicity, each rests on 36 to 88 events, and total cholesterol adds nothing to EuroSCORE 2; we therefore do not build a claim on them. They are nonetheless the part of our data that a purely severity-driven interpretation explains least well, and we prefer to say so rather than present a uniformly negative result.

### Statin therapy

Patients receiving statins had lower TC, LDL-C and HDL-C concentrations, and a nominally significant interaction, which did not survive correction for multiplicity, was found between statin use and the LDL-C-mortality relationship. This observation cannot be attributed to statin therapy itself. Statin-treated patients differ systematically from untreated patients in cardiovascular disease burden and in health-seeking behavior, and observational analyses of statin therapy are subject to confounding by indication, healthy-user bias and healthy-adherer bias, none of which this design can separate. The absence of data on molecule, dose, duration, indication and adherence, and the absence of information on PCSK9 inhibitors, compound this limitation. Our results provide no basis for modifying perioperative lipid-lowering therapy.

### Prognostic association versus prediction

A statistically detectable association and a clinically useful predictor are different things, and preoperative lipid concentrations are the former at most. Areas under the curve between 0.57 and 0.62 are close to chance, and EuroSCORE 2 alone performs substantially better at 0.740. Adding any lipid parameter to EuroSCORE 2 changed discrimination by no more than 0.025, never significantly, with negligible integrated discrimination improvement. Non-HDL-C, an atherogenic-particle metric aligned with current European Atherosclerosis Society thinking,^3^^,^^21^^,^^29^ behaved no differently from LDL-C, which further argues against a lipid-specific mechanism. Preoperative lipid measurement should not be presented, and is not presented here, as a tool for individual risk prediction before cardiac surgery. This does not make the measurement uninformative at the bedside. A particularly low preoperative total cholesterol concentration, in a patient in whom it was not expected, is worth reading as a signal about the patient's general condition rather than about lipid metabolism, and should prompt attention to nutritional status, inflammation and comorbidity rather than to lipid-lowering treatment. We propose no threshold for this, since with 64 events any threshold derived from these data is unstable.

### Mechanistic hypotheses requiring confirmation

None of the mechanisms discussed in this section was measured in the present study, and the following is offered as hypothesis rather than as finding. It is known that in basal non-inflammatory conditions HDL particles have protective effects, but that in inflammatory contexts such as atherosclerosis, sepsis or CPB, HDL particles become dysfunctional, with an altered proteome richer in pro-inflammatory and pro-oxidative proteins,^30^, ^31^, ^32^ and that the raw HDL-C concentration should be complemented by measures of particle functionality to predict outcomes.^33^ Baseline cholesterol concentrations alone may not reflect the prognostic impact of lipid metabolism in patients undergoing CPB, since intraoperative variations may also matter: in a cohort of 192 cardiac surgery patients, Mihalj et al. found a significant intraoperative decrease in HDL-C and a relationship between this concentration and postoperative complications.^18^ Whether lipoprotein function, rather than cholesterol mass, carries prognostic information in this setting is an open question that our data cannot address, and one that will require dedicated mechanistic studies measuring particle composition and functionality.

### Limitations

This study has several limitations. It is a retrospective analysis of a prospectively maintained single-centre registry, and is therefore tied to local practice; the homogeneity of surgical teams and protocols is a partial compensation but not a substitute for external validation.

Only 1,986 of the 10,017 patients remaining after exclusion of early reoperations and duplicate records had a preoperative lipid panel, and the analyzed cohort does not represent the source population: it contains proportionally more valve surgery and cardiac transplantation, fewer isolated CABG procedures, and longer CPB times. We found no imbalance in urgency, endocarditis, critical preoperative state, EuroSCORE 2 or mortality, but unmeasured selection cannot be excluded, and the mechanism of missingness is not verifiable.

With 64 deaths, model complexity was constrained to approximately six covariates, subgroup analyses are underpowered, with nine deaths in the isolated CABG stratum, and the study is adequately powered only for large effects. No a priori sample size calculation was performed, the cohort comprising all eligible patients over the inclusion period. Age was not entered as a separate covariate, since it is a component of EuroSCORE 2.

The INSEE files record the date but not the cause of death, so cause-specific mortality could not be examined for deaths occurring after discharge. Postoperative complications are recorded in the registry as binary items without severity staging, which is why acute kidney injury is less frequent than in series applying KDIGO criteria prospectively, and renal replacement therapy was not recorded in most patients operated on from 2020 onwards, while the hospital discharge date was missing in 22% of patients. Nutritional status could not be characterized: albumin, prealbumin, weight loss and frailty scores are not recorded in the registry, C-reactive protein and total protein were available in only about half of the cohort, and their availability was itself related to the elective care pathway. Whether low lipoprotein concentrations reflect undernutrition, inflammation or both therefore remains a hypothesis that this study cannot resolve, and that would require a prospective evaluation with a dedicated nutritional assessment and measurement of lipoprotein functionality.

The interval between lipid sampling and surgery varied with the clinical context, and the patients sampled on the day of surgery were also the sickest. Restricting the analysis to shorter windows did not change the estimates, and the interval neither predicted mortality nor modified the association, but lipid concentrations change rapidly during acute illness, and a single preoperative measurement cannot capture their trajectory. The change of LDL-C method in 2017 means that the exposure was not measured identically throughout, and since only 26% of the cohort was recruited before that date, period and assay are partly confounded; the estimates were nonetheless similar in the two eras (see Results, the association did not persist after adjustment 3.5). Fasting status was not recorded, which affects triglycerides in particular. Apolipoprotein B and HDL particle functionality were not measured.

## Conclusion

In 1,986 patients undergoing cardiac surgery with CPB, low preoperative TC, HDL-C and LDL-C concentrations were associated with 28-day mortality in unadjusted analysis. After adjustment for markers of illness severity, LDL-C was no longer associated with mortality, and only TC retained a borderline association that did not survive correction for multiplicity. A particularly low preoperative TC should be identified as a marker of underlying illness severity rather than as a causal risk factor. Whether lipoprotein function, rather than cholesterol mass, carries prognostic information in this setting remains an open question.

## Ethics approval statement

The Cardiobase registry was approved on 19 March 2015 by the Comité d'Evaluation de l'Ethique des projets de Recherche Biomédicale (CEERB) Paris Nord (IRB 00006477; project 15-013) and declared to the CNIL (n° 1865399). As a non-interventional study of routinely collected data, written informed consent was not required under French regulations; patients received written information and could object to the use of their data. The approval letter and the ClinicalTrials.gov registration (NCT03393169) are provided as Supplementary Files 1a and 1b.

## Declaration of generative AI and AI-assisted technologies

During the revision of this work, the authors used an AI assistant (Claude, Anthropic) to assist with the coding of sensitivity analyses, and the drafting and language editing of the text. Only anonymized, aggregated data were shared with the tool. The authors reviewed and verified all analyses and all content, and take full responsibility for the manuscript.

## Authors’ contributions

CDT and ST designed the study and methodology. CDT collected the data, curated the database, supervised the project, performed the statistical analyses, and wrote the manuscript. ST conceptualized and supervised the project and wrote the first draft of the manuscript. CE and AB collected the data. PN, MP, BI, OM, SA, DL, PM, and SP critically reviewed and edited the manuscript. All authors read and approved the final manuscript.

## Funding

This research received no specific grant from any funding agency in the public, commercial, or not-for-profit sectors.

## Data availability statement

The data that support the findings of this study are available from the corresponding author upon reasonable request.

## Conflicts of interest

The authors declare no conflicts of interest.
