# Supplementary material for: Preoperative lipid concentrations and 28-day mortality after cardiac surgery with cardiopulmonary bypass: a retrospective analysis of a prospectively maintained registry
Source: Clinics (Sao Paulo). 2026 Nov 3;81:101224. doi: 10.1016/j.clinsp.2026.101224 (PMC13756522; doi:10.1016/j.clinsp.2026.101224)
Supplement: Supplementary file 1 [file mmc1.docx]

**CLINICS-D-26-01385_Supplementary Material**

**Preoperative lipid concentrations and 28-day mortality after cardiac surgery with cardiopulmonary bypass: a retrospective analysis of a prospectively maintained registry**

de Tymowski C, Echard C, Boutten A, Nataf P, Para M, Iung B, Meilhac O, Andrei S, Longrois D, Montravers P, Provenchère S, Tanaka S.

**Contents**

Supplementary Table S1. Comparison of included and excluded patients

Supplementary Table S2. Interval between lipid sampling and surgery: distribution according to preoperative status, and patient characteristics by interval category

Supplementary Table S3. Correction for multiplicity across the primary family of lipid parameters

Supplementary Table S4. Interval between lipid sampling and surgery: sensitivity analyses

Supplementary Table S5. Missing data in the analysed cohort and multiple imputation

Supplementary Table S6. Sensitivity analysis: full-confounder model

Supplementary Table S7. Sensitivity analysis: backward stepwise model

Supplementary Table S8. Mortality, median lipid concentrations and EuroSCORE 2 according to the preoperative condition

Supplementary Table S9. Spearman correlations between preoperative lipid parameters and markers of severity, inflammation, nutrition and organ function

Supplementary Table S10. Availability of severity, nutritional and inflammatory markers, and characteristics according to C-reactive protein measurement

Supplementary Table S11. Sensitivity analysis: models including C-reactive protein and lymphocyte count, every covariate shown

Supplementary Table S12. Secondary outcome: 90-day mortality

Supplementary Figures S1-S9

Appendix A. STROCSS 2025 checklist

Appendix B. STROBE checklist

**Supplementary Table S1 Comparison of included and excluded patients.**

| **Variable** | **Included (n = 1,986)** | **Excluded (n = 8,031)** | **Standardised mean difference** | **p value** |
| --- | --- | --- | --- | --- |
| EuroSCORE 2 (%) | 2.2 [1.2–4.2] | 2.0 [1.1–4.0] | +0.035 | <0.001 |
| Body mass index (kg/m²) | 26.3 [23.5–30.1] | 26.4 [23.8–29.8] | -0.019 | 0.599 |
| Male sex | 1,349 (67.9) | 5,820 (72.5) | -0.099 | <0.001 |
| Arterial hypertension | 1,078 (54.3) | 4,984 (62.1) | -0.158 | <0.001 |
| Diabetes | 525 (26.4) | 2,416 (30.1) | -0.081 | 0.002 |
| Dyslipidaemia | 838 (42.2) | 4,144 (51.6) | -0.189 | <0.001 |
| Active smoking | 422 (21.2) | 1,603 (20.0) | +0.032 | 0.212 |
| COPD | 96 (4.8) | 540 (6.7) | -0.081 | 0.002 |
| Peripheral arterial disease | 184 (9.3) | 879 (10.9) | -0.056 | 0.033 |
| Previous stroke | 185 (9.3) | 746 (9.3) | +0.001 | 1.000 |
| Dialysis-dependent renal failure | 43 (2.2) | 114 (1.4) | +0.056 | 0.022 |
| Heart failure | 206 (10.4) | 502 (6.3) | +0.150 | <0.001 |
| LVEF < 30 % | 124 (6.2) | 371 (4.6) | +0.072 | 0.003 |
| Cirrhosis | 23 (1.2) | 83 (1.0) | +0.012 | 0.716 |
| Ischaemic heart disease | 798 (40.2) | 4,281 (53.3) | -0.265 | <0.001 |
| Prior cardiac surgery | 232 (11.7) | 707 (8.8) | +0.095 | <0.001 |
| Infective endocarditis | 109 (5.5) | 549 (6.8) | -0.056 | 0.034 |
| Urgent surgery | 153 (7.7) | 749 (9.3) | -0.058 | 0.027 |
| Critical preoperative state | 95 (4.8) | 339 (4.2) | +0.027 | 0.298 |
| High-risk preoperative status | 270 (13.6) | 1,230 (15.3) | -0.049 | 0.059 |
| Long-term statin | 948 (47.7) | 4,704 (58.6) | -0.219 | <0.001 |
| ACE inhibitor / ARB | 920 (46.3) | 3,899 (48.6) | -0.045 | 0.079 |
| Beta-blocker | 1,124 (56.6) | 4,728 (58.9) | -0.046 | 0.068 |
| Antiplatelet agent | 917 (46.2) | 4,751 (59.2) | -0.262 | <0.001 |
| Haemoglobin (g/dL) | 13.3 [11.7–14.5] | 13.3 [12.0–14.4] | -0.022 | 0.449 |
| Platelets (G/L) | 221.0 [182.0–269.0] | 220.0 [182.0–266.0] | +0.048 | 0.417 |
| Prothrombin time (%) | 91.0 [81.0–100.0] | 92.0 [82.0–100.0] | -0.050 | 0.034 |
| Creatinine (µmol/L) | 86.0 [73.0–108.0] | 87.0 [74.0–107.0] | +0.045 | 0.674 |
| Isolated CABG | 589 (29.7) | 3,418 (42.6) | -0.271 | <0.001 |
| Valve surgery (alone or combined) | 1,145 (57.7) | 3,840 (47.8) | +0.198 | <0.001 |
| Thoracic aortic surgery (alone or combined, including transplant recipients) | 213 (10.7) | 848 (10.6) | +0.005 | 0.861 |
| Cardiac transplantation | 89 (4.5) | 208 (2.6) | +0.103 | <0.001 |
| CPB duration (min) | 75.0 [55.0–107.0] | 60.0 [46.0–90.0] | +0.273 | <0.001 |
| Cross-clamp duration (min) | 58.0 [43.0–81.0] | 47.0 [37.0–69.0] | +0.293 | <0.001 |
| 28-day mortality | 64 (3.2) | 288 (3.6) | -0.020 | 0.472 |
| 90-day mortality | 88 (4.4) | 395 (4.9) | -0.023 | 0.396 |

Continuous variables are expressed as median [interquartile range]; categorical variables as n (%). Standardised mean differences are computed as the difference in means divided by the pooled standard deviation; an absolute value above 0.10 was considered meaningful. With more than 10,000 patients, p values are reported for completeness only. Valve surgery and thoracic aortic surgery are counted whatever the associated procedure and overlap; Table 1 of the main text presents the type of surgery as mutually exclusive categories.

**Supplementary Table S2 Interval between lipid sampling and surgery: distribution according to preoperative status, and patient characteristics by interval category.**

| **Preoperative status** | **n** | **Median [IQR], days** | **Sampled on the day of surgery** | **Sampled within 1 day** |
| --- | --- | --- | --- | --- |
| Infective endocarditis | 109 | 2 [1–4] | 7.3 % | 34.9 % |
| Critical preoperative state | 82 | 6 [2–11] | 12.2 % | 22.0 % |
| Urgent procedure | 79 | 2 [0–7] | 32.9 % | 46.8 % |
| Stable elective | 1,716 | 3 [1–7] | 2.2 % | 30.7 % |
| **Characteristics by sampling interval** | **n** | **Deaths (%)** | **Median LDL-C (mmol/L)** | **Median HDL-C (mmol/L)** |
| Day of surgery | 82 | 6 (7.3 %) | 2.27 | 1.02 |
| 1-3 days before | 1,017 | 23 (2.3 %) | 2.38 | 1.16 |
| 4-7 days before | 485 | 20 (4.1 %) | 2.57 | 1.07 |
| 8-15 days before | 402 | 15 (3.7 %) | 2.50 | 1.12 |

Upper panel: interval between the lipid panel and surgery according to preoperative status, groups defined as in Supplementary Table S8 (Kruskal–Wallis p < 0.001). Lower panel: patient characteristics and 28-day mortality according to the interval category. The sampling date is that of the panel closest to surgery within the 15-day window; 291 patients had more than one panel in the window, the closest being used. Panels drawn on the day of surgery belong to the most acutely ill patients. Sensitivity analyses on the interval are given in Supplementary Table S4.

**Supplementary Table S3 Correction for multiplicity across the primary family of lipid parameters (prespecified model).**

| **Lipid parameter (per 1 mmol/L)** | **Adjusted OR (95 % CI)** | **Raw p** | **Benjamini–Hochberg q** | **Holm p** | **Bonferroni p** |
| --- | --- | --- | --- | --- | --- |
| Total cholesterol | 0.777 (0.607-0.994) | 0.045 | 0.173 | 0.180 | 0.180 |
| Triglycerides | 0.780 (0.546-1.115) | 0.173 | 0.173 | 0.304 | 0.692 |
| HDL-C | 0.538 (0.261-1.107) | 0.092 | 0.173 | 0.276 | 0.368 |
| LDL-C | 0.803 (0.595-1.084) | 0.152 | 0.173 | 0.304 | 0.608 |

The family comprises the four lipid parameters tested in the prespecified model. The Benjamini–Hochberg q values are identical across the family because the step-up procedure enforces monotonicity, the least significant comparison bounding all others. No correction was applied to exploratory analyses. CI, confidence interval; OR, odds ratio.

**Supplementary Table S4 Interval between lipid sampling and surgery: sensitivity analyses.**

| **Prespecified model restricted to narrower windows** | **n / deaths** | **LDL-C** | **HDL-C** | **TC** | **TG** |
| --- | --- | --- | --- | --- | --- |
| All (≤ 15 days) | 1,970 / 64 | 0.80 (0.59–1.08), 0.152 | 0.54 (0.26–1.11), 0.092 | 0.78 (0.61–0.99), 0.045 | 0.78 (0.55–1.12), 0.173 |
| ≤ 7 days | 1,571 / 49 | 0.74 (0.52–1.06), 0.102 | 0.62 (0.27–1.42), 0.262 | 0.74 (0.55–0.99), 0.043 | 0.82 (0.56–1.20), 0.310 |
| ≤ 3 days | 1,092 / 29 | 0.77 (0.48–1.24), 0.277 | 0.31 (0.10–1.03), 0.056 | 0.72 (0.49–1.06), 0.094 | 0.85 (0.53–1.36), 0.505 |
| ≤ 1 day | 617 / 21 | 0.74 (0.43–1.30), 0.302 | 0.45 (0.12–1.67), 0.231 | 0.71 (0.46–1.10), 0.127 | 0.93 (0.55–1.55), 0.768 |
| ≥ 2 days (excluding day of surgery and day before) | 1,353 / 43 | 0.82 (0.57–1.19), 0.298 | 0.58 (0.24–1.40), 0.226 | 0.80 (0.59–1.08), 0.151 | 0.66 (0.40–1.10), 0.112 |

Adjusted odds ratios per 1 mmol/L increase (95 % CI) and p values, from the prespecified model refitted after restriction to narrower sampling windows. n and deaths refer to patients with an available LDL-C value; the TC, HDL-C and TG models additionally include the patients without LDL-C (1,986 in the full window). Entered as a covariate in the LDL-C model, the interval was not associated with mortality (odds ratio 1.017 per day, 95 % CI 0.954-1.085, p = 0.602) and did not modify the association of LDL-C (interaction p = 0.160) or HDL-C (p = 0.786) with mortality. CI, confidence interval.

**Supplementary Table S5 Missing data in the analysed cohort (n = 1,986) and multiple imputation.**

| **Variable** | **Missing, n** | **Missing, %** |
| --- | --- | --- |
| 28-day mortality | 0 | 0.0 |
| Total cholesterol | 0 | 0.0 |
| HDL-C | 0 | 0.0 |
| LDL-C | 16 | 0.8 |
| Triglycerides | 0 | 0.0 |
| EuroSCORE 2 | 0 | 0.0 |
| Long-term statin | 0 | 0.0 |
| Infective endocarditis | 0 | 0.0 |
| Urgent surgery | 0 | 0.0 |
| Critical preoperative state | 0 | 0.0 |
| Heart failure | 0 | 0.0 |
| Dialysis-dependent renal failure | 0 | 0.0 |
| Cirrhosis | 0 | 0.0 |
| Haemoglobin | 38 | 1.9 |
| Prothrombin time | 121 | 6.1 |
| CPB duration | 46 | 2.3 |
| Lymphocyte count | 690 | 34.7 |
| C-reactive protein | 824 | 41.5 |
| Total protein | 871 | 43.9 |
| Pooled estimates after multiple imputation (m = 20), prespecified model: OR (95 % CI) and p value |  |  |
| Total cholesterol (per 1 mmol/L) | 0.777 (0.607–0.994) | 0.045 |
| HDL-C (per 1 mmol/L) | 0.538 (0.261–1.107) | 0.092 |
| LDL-C (per 1 mmol/L) | 0.803 (0.595–1.085) | 0.153 |

Upper panel: number and proportion of missing values for each analysis variable within the analysed cohort. Lower panel: pooled adjusted odds ratios (95 % CI) for 28-day mortality after multiple imputation by chained equations (20 imputations, 10 iterations). Within the analysed cohort, no covariate of the prespecified model was missing, so that only the 16 missing LDL-C values were imputed; the exposure was not imputed in excluded patients. CI, confidence interval; OR, odds ratio.

**Supplementary Table S6 Sensitivity analysis: full-confounder model, adjusted odds ratios (95 % CI) for every covariate, one model per lipid parameter.**

| **Covariate** | **TC** | **TG** | **HDL-C** | **LDL-C** |
| --- | --- | --- | --- | --- |
| Lipid parameter (per 1 mmol/L) | 0.820 (0.640-1.050)  p = 0.116 | 0.766 (0.529-1.109)  p = 0.158 | 0.600 (0.291-1.237)  p = 0.166 | 0.861 (0.636-1.164)  p = 0.330 |
| EuroSCORE 2 (per point) | 1.090 (1.054-1.127)  p < 0.001 | 1.087 (1.051-1.124)  p < 0.001 | 1.093 (1.056-1.131)  p < 0.001 | 1.088 (1.052-1.125)  p < 0.001 |
| Long-term statin therapy | 0.728 (0.407-1.303)  p = 0.285 | 0.837 (0.474-1.478)  p = 0.540 | 0.776 (0.439-1.372)  p = 0.383 | 0.744 (0.413-1.341)  p = 0.326 |
| Infective endocarditis | 1.597 (0.698-3.654)  p = 0.267 | 1.905 (0.845-4.294)  p = 0.120 | 1.488 (0.628-3.528)  p = 0.367 | 1.735 (0.762-3.948)  p = 0.189 |
| Urgent surgery | 1.557 (0.680-3.566)  p = 0.295 | 1.726 (0.766-3.887)  p = 0.188 | 1.601 (0.704-3.642)  p = 0.261 | 1.621 (0.711-3.698)  p = 0.251 |
| Critical preoperative state | 0.488 (0.152-1.565)  p = 0.228 | 0.555 (0.173-1.780)  p = 0.322 | 0.463 (0.141-1.522)  p = 0.205 | 0.527 (0.165-1.680)  p = 0.279 |
| Heart failure | 0.920 (0.417-2.029)  p = 0.837 | 0.906 (0.409-2.007)  p = 0.807 | 0.899 (0.403-2.002)  p = 0.794 | 0.946 (0.429-2.086)  p = 0.891 |
| Dialysis-dependent renal failure | 3.567 (1.231-10.336)  p = 0.019 | 4.175 (1.453-11.996)  p = 0.008 | 3.782 (1.321-10.831)  p = 0.013 | 3.656 (1.258-10.626)  p = 0.017 |
| Cirrhosis | 3.101 (0.773-12.441)  p = 0.110 | 3.675 (0.938-14.403)  p = 0.062 | 3.235 (0.801-13.057)  p = 0.099 | 3.320 (0.832-13.246)  p = 0.089 |
| Redo surgery | 1.020 (0.480-2.167)  p = 0.958 | 1.043 (0.491-2.215)  p = 0.912 | 1.037 (0.487-2.208)  p = 0.925 | 1.032 (0.486-2.192)  p = 0.935 |
| n | 1,986 | 1,986 | 1,986 | 1,970 |

The prespecified model further adjusted for heart failure, dialysis-dependent renal failure, cirrhosis and redo surgery. Sex is a component of EuroSCORE 2 and was not entered separately. With 64 events this model exceeds the events-per-variable rule and is reported as a sensitivity analysis only; the estimates for the lipid parameters are unchanged with respect to the prespecified model. Values in bold: p < 0.05. CI, confidence interval; HDL-C, high-density lipoprotein cholesterol; LDL-C, low-density lipoprotein cholesterol; TC, total cholesterol; TG, triglycerides.

**Supplementary Table S7 Sensitivity analysis: backward stepwise model, adjusted odds ratios (95 % CI) for every retained covariate, one model per lipid parameter.**

| **Covariate retained** | **TC** | **TG** | **HDL-C** | **LDL-C** |
| --- | --- | --- | --- | --- |
| Lipid parameter (per 1 mmol/L) | 0.823 (0.650-1.040)  p = 0.103 | 0.762 (0.528-1.100)  p = 0.147 | 0.581 (0.296-1.141)  p = 0.115 | not retained |
| EuroSCORE 2 (per point) | 1.089 (1.066-1.113)  p < 0.001 | 1.084 (1.059-1.110)  p < 0.001 | 1.090 (1.067-1.114)  p < 0.001 | 1.086 (1.061-1.112)  p < 0.001 |
| Infective endocarditis | not retained | 1.957 (0.881-4.345)  p = 0.099 | not retained | 1.910 (0.860-4.243)  p = 0.112 |
| Dialysis-dependent renal failure | 3.279 (1.136-9.467)  p = 0.028 | 3.890 (1.358-11.145)  p = 0.011 | 3.458 (1.209-9.893)  p = 0.021 | 3.738 (1.317-10.607)  p = 0.013 |
| Cirrhosis | 3.177 (0.824-12.248)  p = 0.093 | 3.430 (0.883-13.318)  p = 0.075 | 3.182 (0.807-12.542)  p = 0.098 | 3.412 (0.867-13.423)  p = 0.079 |
| n | 1,986 | 1,986 | 1,986 | 1,970 |

Backward selection on the Akaike information criterion, applied to the same candidate set as the full-confounder model. "Not retained" indicates that the variable was dropped during selection. The lipid parameter is retained for TC, TG and HDL-C but not for LDL-C, and in no model does it reach the 0.05 threshold. That the variable retained differs across models illustrates the instability of stepwise selection with 64 events, which is why the primary model is prespecified rather than selected. Values in bold: p < 0.05. CI, confidence interval.

**Supplementary Table S8 Mortality, median lipid concentrations and EuroSCORE 2 according to the preoperative condition.**

| **Group** | **n** | **Deaths** | **28-day mortality** | **TC (mmol/L)** | **TG (mmol/L)** | **HDL-C (mmol/L)** | **LDL-C (mmol/L)** | **EuroSCORE 2 (%)** |
| --- | --- | --- | --- | --- | --- | --- | --- | --- |
| Infective endocarditis | 109 | 13 | 11.9 % | 3.28 | 1.33 | 0.74 | 2.04 | 4.19 |
| Critical preoperative state | 82 | 8 | 9.8 % | 3.19 | 1.12 | 0.90 | 2.02 | 12.71 |
| Urgent procedure | 79 | 7 | 8.9 % | 3.69 | 1.24 | 0.99 | 2.23 | 3.74 |
| Stable elective | 1,716 | 36 | 2.1 % | 4.23 | 1.37 | 1.17 | 2.51 | 1.95 |
| Kruskal–Wallis p value |  |  |  | <0.001 | 0.013 | <0.001 | <0.001 |  |

Mutually exclusive groups (Methods 2.4): a patient meeting more than one criterion is classed in the first applicable group, in the order infective endocarditis, critical preoperative state, urgent procedure. Lipid concentrations are medians; p values from the Kruskal–Wallis test. HDL-C, high-density lipoprotein cholesterol; LDL-C, low-density lipoprotein cholesterol; TC, total cholesterol; TG, triglycerides.

**Supplementary Table S9 Spearman correlations between preoperative lipid parameters and markers of severity, inflammation, nutrition and organ function.**

| **Marker** | **Total cholesterol, ρ (p)** | **HDL-C, ρ (p)** | **LDL-C, ρ (p)** | **n (LDL-C)** |
| --- | --- | --- | --- | --- |
| Lymphocyte count | +0.109 (<0.001) | -0.087 (0.002) | +0.124 (<0.001) | 1,280 |
| Neutrophil-to-lymphocyte ratio | -0.183 (<0.001) | -0.114 (<0.001) | -0.172 (<0.001) | 1,280 |
| C-reactive protein | -0.091 (0.002) | -0.251 (<0.001) | -0.032 (0.283) | 1,150 |
| Total protein | +0.154 (<0.001) | +0.041 (0.167) | +0.153 (<0.001) | 1,100 |
| Haemoglobin | +0.212 (<0.001) | +0.091 (<0.001) | +0.203 (<0.001) | 1,933 |
| Prothrombin time | +0.228 (<0.001) | +0.230 (<0.001) | +0.130 (<0.001) | 1,851 |

Correlations are weak throughout (largest absolute rho 0.25, all others below 0.24). The strongest is the inverse correlation between HDL-C and C-reactive protein. Availability differs across markers; n is given for the LDL-C column.

**Supplementary Table S10 Availability of severity, nutritional and inflammatory markers, and characteristics according to C-reactive protein measurement.**

| **Marker** | **n available** | **% of the cohort** |
| --- | --- | --- |
| Haemoglobin | 1,948 | 98.1 % |
| Creatinine | 1,947 | 98.0 % |
| Prothrombin time | 1,865 | 93.9 % |
| Lymphocyte count | 1,296 | 65.3 % |
| Neutrophil-to-lymphocyte ratio | 1,296 | 65.3 % |
| C-reactive protein | 1,162 | 58.5 % |
| Total protein | 1,115 | 56.1 % |

Upper panel: number and proportion of patients in whom each marker was available within the analysed cohort (n = 1,986). Albumin could not be retrieved from the institutional database. Characteristics according to C-reactive protein measurement are given below.

|  | **n** | **Urgent surgery** | **Infective endocarditis** | **Critical preoperative state** | **Median EuroSCORE 2** | **28-day mortality** |
| --- | --- | --- | --- | --- | --- | --- |
| CRP measured | 1,162 | 3.9 % | 4.4 % | 2.3 % | 2.07 | 3.0 % |
| CRP not measured | 824 | 13.1 % | 7.0 % | 8.3 % | 2.32 | 3.5 % |

C-reactive protein is drawn along the elective work-up pathway: patients in whom it was measured had a lower prevalence of urgent surgery, infective endocarditis and critical preoperative state than those in whom it was not. A model adjusted for C-reactive protein therefore excludes the acutely ill patients who carry the association, which is why it is reported as a sensitivity analysis only (Supplementary Table S11).

**Supplementary Table S11 Sensitivity analysis: models including C-reactive protein and lymphocyte count, adjusted odds ratios (95 % CI) for every covariate, one model per lipid parameter, in the subgroup in which both markers were available.**

| **Covariate** | **TC** | | **HDL-C** | | **LDL-C** | |
| --- | --- | --- | --- | --- | --- | --- |
|  | **Model 1** | **Model 2** | **Model 1** | **Model 2** | **Model 1** | **Model 2** |
| Lipid parameter (per 1 mmol/L) | 0.883 (0.639–1.221)  p = 0.452 | 0.960 (0.693–1.329)  p = 0.804 | 0.424 (0.155–1.160)  p = 0.095 | **0.358 (0.131–0.978)**  **p = 0.045** | 0.936 (0.631–1.387)  p = 0.741 | 1.066 (0.717–1.586)  p = 0.751 |
| EuroSCORE 2 (per point) | **1.101 (1.055–1.150)**  **p < 0.001** | **1.092 (1.046–1.139)**  **p < 0.001** | **1.110 (1.062–1.159)**  **p < 0.001** | **1.102 (1.055–1.151)**  **p < 0.001** | **1.102 (1.054–1.151)**  **p < 0.001** | **1.092 (1.046–1.140)**  **p < 0.001** |
| Long-term statin therapy | 0.597 (0.278–1.284)  p = 0.187 | 0.621 (0.287–1.343)  p = 0.226 | 0.609 (0.286–1.294)  p = 0.197 | 0.626 (0.291–1.346)  p = 0.231 | 0.614 (0.281–1.338)  p = 0.220 | 0.650 (0.297–1.425)  p = 0.282 |
| Infective endocarditis | 1.515 (0.446–5.144)  p = 0.506 | 1.013 (0.285–3.608)  p = 0.984 | 1.090 (0.298–3.992)  p = 0.897 | 0.618 (0.159–2.406)  p = 0.488 | 1.634 (0.481–5.553)  p = 0.431 | 1.067 (0.304–3.750)  p = 0.919 |
| Urgent surgery | 0.345 (0.054–2.204)  p = 0.261 | 0.278 (0.045–1.707)  p = 0.167 | 0.322 (0.050–2.081)  p = 0.234 | 0.270 (0.047–1.570)  p = 0.145 | 0.349 (0.055–2.230)  p = 0.266 | 0.271 (0.044–1.684)  p = 0.161 |
| Critical preoperative state | 1.002 (0.194–5.182)  p = 0.998 | 0.953 (0.199–4.562)  p = 0.952 | 0.795 (0.149–4.235)  p = 0.788 | 0.673 (0.133–3.398)  p = 0.632 | 1.023 (0.197–5.325)  p = 0.978 | 0.926 (0.192–4.453)  p = 0.923 |
| C-reactive protein (per 10 mg/L) | — | 1.053 (0.976–1.136)  p = 0.182 | — | 1.046 (0.968–1.131)  p = 0.256 | — | 1.061 (0.982–1.147)  p = 0.134 |
| Lymphocyte count (per G/L) | — | **0.344 (0.182–0.647)**  **p < 0.001** | — | **0.315 (0.167–0.595)**  **p < 0.001** | — | **0.341 (0.180–0.645)**  **p < 0.001** |
| n / deaths | 1,159 / 35 | 1,159 / 35 | 1,159 / 35 | 1,159 / 35 | 1,147 / 35 | 1,147 / 35 |

Model 1 is the prespecified model refitted in the subgroup with available C-reactive protein and lymphocyte count; Model 2 adds C-reactive protein (per 10 mg/L) and lymphocyte count (per G/L). Values in bold: p < 0.05. CI, confidence interval; HDL-C, high-density lipoprotein cholesterol; LDL-C, low-density lipoprotein cholesterol; TC, total cholesterol.

**Supplementary Table S12 Secondary outcome: 90-day mortality, adjusted odds ratios (95 % CI) for every covariate, one model per lipid parameter.**

| **Covariate** | **TC** | **TG** | **HDL-C** | **LDL-C** |
| --- | --- | --- | --- | --- |
| Lipid parameter (per 1 mmol/L) | 0.799 (0.649-0.984)  p = 0.035 | 0.803 (0.597-1.082)  p = 0.150 | 0.637 (0.348-1.167)  p = 0.144 | 0.811 (0.627-1.049)  p = 0.110 |
| EuroSCORE 2 (per point) | 1.092 (1.064-1.122)  p < 0.001 | 1.090 (1.061-1.120)  p < 0.001 | 1.096 (1.066-1.126)  p < 0.001 | 1.091 (1.062-1.121)  p < 0.001 |
| Long-term statin therapy | 0.801 (0.493-1.302)  p = 0.372 | 0.930 (0.579-1.493)  p = 0.763 | 0.867 (0.540-1.394)  p = 0.557 | 0.828 (0.505-1.358)  p = 0.455 |
| Infective endocarditis | 1.223 (0.568-2.635)  p = 0.607 | 1.521 (0.717-3.227)  p = 0.274 | 1.200 (0.540-2.667)  p = 0.655 | 1.348 (0.627-2.898)  p = 0.445 |
| Urgent surgery | 1.381 (0.658-2.896)  p = 0.393 | 1.516 (0.731-3.144)  p = 0.263 | 1.440 (0.690-3.006)  p = 0.332 | 1.437 (0.685-3.012)  p = 0.337 |
| Critical preoperative state | 0.648 (0.252-1.665)  p = 0.368 | 0.770 (0.304-1.951)  p = 0.582 | 0.630 (0.241-1.650)  p = 0.347 | 0.722 (0.284-1.840)  p = 0.496 |
| n / deaths | 1,986 / 88 | 1,986 / 88 | 1,986 / 88 | 1,970 / 87 |

Same prespecified model as for 28-day mortality, applied to 90-day all-cause mortality (88 deaths). Total cholesterol is nominally associated with 90-day mortality (odds ratio 0.799 per 1 mmol/L, 95 % CI 0.649-0.984, p = 0.035), as it is at 28 days (0.777, 0.607-0.994, p = 0.045); this association does not survive correction for multiplicity across the four lipid parameters (Benjamini–Hochberg q = 0.140 at 90 days; Supplementary Table S3 for 28 days). No other lipid parameter is associated with 90-day mortality, and EuroSCORE 2 remains the only other covariate associated with the outcome. Values in bold: p < 0.05. CI, confidence interval; HDL-C, high-density lipoprotein cholesterol; LDL-C, low-density lipoprotein cholesterol; TC, total cholesterol; TG, triglycerides.

**Supplementary Figures**

**Supplementary Figure S1 Temporal trends over the study period.**

(A) Proportion of the analysed cohort operated on in each two-year period (2021–2023 forming the last period). (B) 28-day mortality by two-year period (Cochran–Armitage trend test). (C–F) Preoperative total cholesterol, triglycerides, HDL-C and LDL-C by two-year period; boxes show the median and interquartile range; p values from the Kruskal–Wallis test. The triglyceride axis is truncated at 8 mmol/L. (G) Proportion of all operations with cardiopulmonary bypass in which a preoperative lipid panel was available, by year of surgery.

**Supplementary Figure S2 Preoperative total cholesterol (A), triglycerides (B), HDL-C (C) and LDL-C (D) according to age.**

Age groups are quintiles of age. Boxes show the median and interquartile range; p values from the Kruskal–Wallis test. The triglyceride axis is truncated at 8 mmol/L.

**Supplementary Figure S3 Preoperative lipid concentrations according to sex.**

Boxes show the median and interquartile range; p values from the Mann–Whitney U test. The triglyceride axis is truncated at 8 mmol/L.

**Supplementary Figure S4 Directed acyclic graph representing the assumed causal structure.**

The minimal sufficient adjustment set for the total effect of preoperative cholesterol on 28-day mortality comprises proxies of illness severity and comorbidity together with statin use, operationalised as EuroSCORE 2, long-term statin use, infective endocarditis, urgent surgery and critical preoperative state. CPB duration lies downstream of surgical complexity and is measured after the exposure; under this structure it is not a confounder of the cholesterol–mortality relationship and is therefore excluded from the adjustment set. CPB, cardiopulmonary bypass.

**Supplementary Figure S5 Proportion of missing values for each analysis variable within the analysed cohort.**

**Supplementary Figure S6 Receiver operating characteristic curves for 28-day mortality.**

(A) Each preoperative lipid parameter alone, including non-HDL-C. (B) EuroSCORE 2 alone (black) and EuroSCORE 2 plus each lipid parameter. For each lipid parameter, mortality is associated with the low value, which is therefore taken as the positive criterion. Areas under the curve are given with their 95 % confidence intervals.

**Supplementary Figure S7 Calibration of the EuroSCORE 2 model with and without LDL-C, by decile of predicted risk.**

Points show observed versus predicted 28-day mortality within each decile of predicted risk; the dashed line is the identity.

**Supplementary Figure S8 Preoperative total cholesterol (A), triglycerides (B), HDL-C (C) and LDL-C (D) according to the preoperative condition.**

Groups as defined in Supplementary Table S8. Boxes show the median and interquartile range; p values from the Kruskal–Wallis test.

**Supplementary Figure S9 Odds ratio for 28-day mortality per 1 mmol/L increase in preoperative LDL-C, overall and by subgroup.**

Each estimate is adjusted for EuroSCORE 2 only, since with 9 to 55 deaths per stratum the full prespecified set could not be fitted in every stratum, and is shown with its 95 % confidence interval, the number of patients with an available LDL-C value and the number of deaths in the stratum; the estimates for the stable elective and statin-free strata under the prespecified set are given in Results 3.8. Subgroup analyses are exploratory. In the isolated CABG and statin strata the confidence interval excludes 1; the corresponding likelihood ratio interaction tests were nominally significant (p = 0.012 and 0.013) but did not survive Benjamini–Hochberg correction across the twelve tests performed (q = 0.062), and the isolated CABG estimate rests on nine deaths.

**Appendix A. STROCSS 2025 checklist**

Agha et al., Premier Journal of Science 2025;10:100081

| **Topic** | **Item** | **Description** | **Location in the revised manuscript** |
| --- | --- | --- | --- |
| Title | 1 | Study design, population and focus identified in the title | Title (retrospective analysis of a prospectively maintained registry; preoperative lipid concentrations; 28-day mortality; cardiac surgery with CPB) |
| Highlights | 2 | 3–5 bullet points summarising the study | Highlights (separate file) |
| Abstract | 3a | Structured abstract | Abstract |
| Abstract | 3b | Background and aims | Abstract, Background |
| Abstract | 3c | Methods overview | Abstract, Methods |
| Abstract | 3d | Results summary | Abstract, Results |
| Abstract | 3e | Conclusion | Abstract, Conclusions |
| Keywords | 4 | Keywords provided | Key words (title page) |
| Artificial intelligence | 5 | Declaration of whether AI was used | Declaration of generative AI and AI-assisted technologies (after the Author Contribution Statement) |
| Artificial intelligence | 5a–5f | Purpose and scope, tool identification, data safeguards, human oversight, bias and ethics, reproducibility | Declaration of generative AI and AI-assisted technologies (after the Author Contribution Statement) |
| Introduction | 6a | Background, rationale and aims | Introduction, paragraphs 1–5 |
| Introduction | 6b | Statement of adherence to STROCSS | Methods 2.5, last paragraph |
| Study design | 7a | Type of study and design | Methods 2.1 |
| Study design | 7b | Setting, centre and study period | Methods 2.1 and 2.2 |
| Study design | 7c | Study groups, eligibility and exclusion criteria | Methods 2.1; Figure 1 |
| Study design | 7d | Subgroups and their definition | Methods 2.4; Results 3.8 |
| Study design | 7e | Follow-up and data sources for outcomes | Methods 2.2 (registry vital status completed with INSEE records) |
| Participant recruitment | 8a | Recruitment period and method | Methods 2.1; Results 3.1 |
| Participant recruitment | 8b | Sample size and its rationale | Methods 2.1 (all eligible patients); Discussion 4.6 (no a priori calculation; 64 events) |
| Intervention and outcomes | 9a–9g | Pre-intervention considerations, intervention details, operator, quality control | Not applicable (non-interventional study of preoperative measurements) |
| Intervention and outcomes | 9h | Primary and secondary outcomes, measurement and timing | Methods 2.4 |
| Intervention and outcomes | 9i | Statistical methods, confounding, missing data, sensitivity analyses | Methods 2.5; Supplementary Tables S1–S7; Supplementary Figures S4 and S5 |
| Results | 10a | Participant flow and numbers at each stage | Results 3.1; Figure 1; Supplementary Table S1 |
| Results | 10b | Baseline characteristics | Table 1 |
| Results | 10c | Outcome data and estimates with precision | Results 3.3–3.9; Tables 2–6; Figures 2–5 |
| Results | 10d | Tolerance and adherence to intervention | Not applicable |
| Results | 10e | Complications and adverse events | Table 2 (postoperative course) |
| Results | 10f | Key results | Results 3.4–3.6; Discussion, first paragraph |
| Discussion | 11a | Principal findings in the context of existing literature | Discussion 4.1–4.2 |
| Discussion | 11b | Strengths and limitations | Discussion 4.6 |
| Discussion | 11c | Implications and generalisability | Discussion 4.2–4.4 and 4.6 |
| Conclusion | 12 | Conclusion consistent with the objectives | Conclusion |
| Additional information | 13a | Registration of the study | Methods 2.2 (ClinicalTrials.gov NCT03393169) |
| Additional information | 13b | Ethical approval | Methods 2.2; Ethics Approval Statement; Supplementary File 1a |
| Additional information | 13c | Consent | Methods 2.2 (non-opposition procedure); Ethics Approval Statement |
| Additional information | 13d | Protocol availability | Not applicable (registry study; analysis plan not registered, stated in Methods 2.4) |
| Declarations | 14a | Author contributions | Author Contribution Statement |
| Declarations | 14b | Conflicts of interest | Conflict of Interest Statement |
| Declarations | 14c | Funding | Funding Statement |
| Declarations | 14d | Data availability | Data Availability Statement |

Locations refer to the numbered sections, tables and figures of the revised manuscript.

**Appendix B. STROBE checklist for cohort studies**

von Elm et al., Lancet 2007;370:1453-1457

| **Item** | **No.** | **Recommendation** | **Location in the revised manuscript** |
| --- | --- | --- | --- |
| Title and abstract | 1 | (a) Indicate the study design with a commonly used term in the title or the abstract; (b) provide an informative and balanced summary | Title; Abstract |
| Background/rationale | 2 | Explain the scientific background and rationale | Introduction, paragraphs 1–4 |
| Objectives | 3 | State specific objectives, including any prespecified hypotheses | Introduction, last paragraph; Methods 2.4 |
| Study design | 4 | Present key elements of study design early in the paper | Methods 2.1 |
| Setting | 5 | Describe the setting, locations, and relevant dates | Methods 2.1–2.3 |
| Participants | 6 | (a) Eligibility criteria, sources and methods of selection, follow-up | Methods 2.1–2.2; Figure 1 |
| Variables | 7 | Define all outcomes, exposures, predictors, potential confounders and effect modifiers | Methods 2.3–2.5; Supplementary Figure S4 |
| Data sources/measurement | 8 | Sources of data and details of methods of assessment | Methods 2.2–2.3 |
| Bias | 9 | Describe any efforts to address potential sources of bias | Methods 2.5 (DAG, standardised differences); Discussion 4.2–4.3 |
| Study size | 10 | Explain how the study size was arrived at | Methods 2.1; Discussion 4.6 (no a priori calculation; all eligible patients) |
| Quantitative variables | 11 | Explain how quantitative variables were handled in the analyses | Methods 2.5 (continuous splines; quintiles exploratory) |
| Statistical methods | 12 | (a) All statistical methods, including confounding; (b) subgroups and interactions; (c) missing data; (d) loss to follow-up; (e) sensitivity analyses | Methods 2.5; Supplementary Tables S3–S7 |
| Participants | 13 | Numbers at each stage; reasons for non-participation; flow diagram | Results 3.1; Figure 1 |
| Descriptive data | 14 | Characteristics of participants; missing data; follow-up time | Table 1; Supplementary Table S5 |
| Outcome data | 15 | Numbers of outcome events | Results 3.1, 3.8 and 3.9; Table 2; Supplementary Figure S9 |
| Main results | 16 | (a) Unadjusted and adjusted estimates with precision; confounders adjusted for; (b) category boundaries; (c) absolute risk | Table 3; Figure 3 (absolute risk by quintile); Figure 4 (adjusted curves); Supplementary Figure S7 (calibration) |
| Other analyses | 17 | Subgroups, interactions, sensitivity analyses | Results 3.5–3.9; Supplementary Tables S1–S12; Supplementary Figure S9 |
| Key results | 18 | Summarise key results with reference to study objectives | Discussion, first paragraph |
| Limitations | 19 | Limitations, sources of potential bias or imprecision, direction and magnitude of bias | Discussion 4.6 |
| Interpretation | 20 | Cautious overall interpretation considering objectives, limitations, multiplicity, similar studies | Discussion 4.1–4.4; Conclusion |
| Generalisability | 21 | Discuss the generalisability (external validity) | Discussion 4.6 |
| Funding | 22 | Source of funding and role of the funders | Funding Statement |

Locations refer to the numbered sections, tables and figures of the revised manuscript.
